# Fibroblast growth factor receptor 4 promotes glioblastoma progression: a central role of integrin-mediated cell invasiveness

**DOI:** 10.1186/s40478-022-01363-2

**Published:** 2022-04-28

**Authors:** Lisa Gabler, Carola Nadine Jaunecker, Sonja Katz, Sushilla van Schoonhoven, Bernhard Englinger, Christine Pirker, Thomas Mohr, Petra Vician, Mirjana Stojanovic, Valentin Woitzuck, Anna Laemmerer, Dominik Kirchhofer, Lisa Mayr, Mery LaFranca, Friedrich Erhart, Sarah Grissenberger, Andrea Wenninger-Weinzierl, Caterina Sturtzel, Barbara Kiesel, Alexandra Lang, Brigitte Marian, Bettina Grasl-Kraupp, Martin Distel, Julia Schüler, Johannes Gojo, Michael Grusch, Sabine Spiegl-Kreinecker, Daniel J. Donoghue, Daniela Lötsch, Walter Berger

**Affiliations:** 1 Center for Cancer Research, Medical University of Vienna, Borschkegasse 8A, 1090 Vienna, Austria; 2 Comprehensive Cancer Center-Central Nervous System Tumor Unit, Medical University of Vienna, Spitalgasse 23, 1090 Vienna, Austria; 3 Department of Neurosurgery, Medical University of Vienna, Waehringer Guertel 18-20, 1090 Vienna, Austria; 4 Department of Pediatrics and Adolescent Medicine and Comprehensive Center for Pediatrics, Medical University of Vienna, Waehringer Guertel 18-20, 1090 Vienna, Austria; 5 Department of Pediatric Oncology, Dana-Farber Boston Children’s Cancer and Blood Disorders Center, Boston, MA 02215 USA; 6 Broad Institute of Harvard and MIT, Cambridge, MA 02142 USA; 7 Department of Biological, Chemical and Pharmaceutical Sciences and Technologies, (STEBICEF), University of Palermo, via Archirafi 32, 90123 Palermo, Italy; 8 St. Anna Children’s Cancer Research Institute, Vienna, Austria; 9 Charles River Discovery Research Services Germany GmbH, Freiburg, Germany; 10 Department of Neurosurgery, Kepler University Hospital GmbH, Johannes Kepler University Linz, Wagner-Jauregg-Weg 15, 4020, Linz and Altenberger Strasse 69, 4020, Linz, Austria; 11 Department of Chemistry and Biochemistry, Moores UCSD Cancer Center, University of California San Diego, La Jolla, CA 92093-0367 USA

**Keywords:** Glioblastoma, FGFR4, Integrins, Invasiveness, FAK, FGF19

## Abstract

Glioblastoma (GBM) is characterized by a particularly invasive phenotype, supported by oncogenic signals from the fibroblast growth factor (FGF)/ FGF receptor (FGFR) network. However, a possible role of FGFR4 remained elusive so far. Several transcriptomic glioma datasets were analyzed. An extended panel of primary surgical specimen-derived and immortalized GBM (stem)cell models and original tumor tissues were screened for FGFR4 expression. GBM models engineered for wild-type and dominant-negative FGFR4 overexpression were investigated regarding aggressiveness and xenograft formation. Gene set enrichment analyses of FGFR4-modulated GBM models were compared to patient-derived datasets. Despite widely absent in adult brain, *FGFR4* mRNA was distinctly expressed in embryonic neural stem cells and significantly upregulated in glioblastoma. Pronounced *FGFR4* overexpression defined a distinct GBM patient subgroup with dismal prognosis. Expression levels of *FGFR4* and its specific ligands *FGF19/FGF23* correlated both in vitro and in vivo and were progressively upregulated in the vast majority of recurrent tumors. Based on overexpression/blockade experiments in respective GBM models, a central pro-oncogenic function of FGFR4 concerning viability, adhesion, migration, and clonogenicity was identified. Expression of dominant-negative FGFR4 resulted in diminished (subcutaneous) or blocked (orthotopic) GBM xenograft formation in the mouse and reduced invasiveness in zebrafish xenotransplantation models. In vitro and in vivo data consistently revealed distinct FGFR4 and integrin/extracellular matrix interactions. Accordingly, FGFR4 blockade profoundly sensitized FGFR4-overexpressing GBM models towards integrin/focal adhesion kinase inhibitors. Collectively, *FGFR4* overexpression contributes to the malignant phenotype of a highly aggressive GBM subgroup and is associated with integrin-related therapeutic vulnerabilities.

**Supplementary Information:**

The online version contains supplementary material available at 10.1186/s40478-022-01363-2.

## Introduction

Glioblastoma (GBM) represents the most common malignant brain tumor in adults [1]. First-line therapy comprising surgical debulking followed by concomitant radio-chemotherapy with temozolomide [2] results in a five-year survival rate of only ~ 6.8% [1]. Besides high invasiveness, GBM is generally characterized by pronounced heterogeneity and cell plasticity, explaining why frequently neither surgical nor pharmacological intervention results in patient cure [3]. Hence, there is an urgent need to dissect oncogenic GBM programs allowing development of novel, integrative treatment perspectives.

The fibroblast growth factor receptor (FGFR) family comprises four highly conserved receptor homologs named FGFR1-4. Out of the 18 known fibroblast growth factors (FGFs) in humans, members of the FGF19 subfamily, including FGF19, FGF21, and FGF23, bind with higher affinity to FGFR4 than to other family members [4]. FGFRs have emerged as potential cancer targets, as overexpression and hyperactivation have been described in a wide array of cancer types [5, 6]. Regarding *FGFR3,* and in rare cases also *FGFR1*, oncogenic chromosomal fusions to *transforming acidic coiled-coil (TACC) 3* or *TACC1,* respectively*,* have been identified in ~ 3% of GBM [7] and related to tumor initiation and progression [8–10]. Compared to other FGFR family members, genetic aberrations of *FGFR4* are rather rare [4, 11]. Instead, a single nucleotide polymorphism (SNP) at codon 388 of *FGFR4* (rs351855), leading to a glycine to arginine conversion (G388R), has been connected to enhanced tumor susceptibility and aggressiveness in different tumor entities [12]. Concerning GBM, one earlier study did not find an impact of the G388R SNP on GBM patient prognosis, despite enhanced *FGFR4* gene expression in case of the 388Arg allele [13]. Nevertheless, a driving role of FGFR4 in astrocytoma malignancy has been suggested, supported by a correlation of FGFR4 protein expression with malignant progression [14]. However, functional studies on FGFR4 in GBM are missing yet.

Here, we identify FGFR4 overexpression in a highly aggressive GBM subgroup and discover a key contribution to the malignant phenotype, especially concerning cell adhesion and migration via an integrin-mediated mechanism. This warrants further investigations of FGFR4 as therapeutic target in this disease, for which currently no targeted agents have reached worldwide clinical approval.

## Materials and methods

### Availability of transcriptomic datasets

*FGFR4* expression in non-malignant tissues was analyzed in the GTEx dataset and the Allen brain atlas (brain-map.org). Data on glioma were derived from the REMBRANDT and TCGA-GBM datasets. Detailed information on data processing and visualization are given in the supplementary materials.

### mRNA expression microarray and array comparative genomic hybridization (aCGH)

Analyses of whole genome gene expression (4 × 44 K microarrays, Agilent Technologies, Santa Clara, USA) and copy number changes by aCGH (4 × 44 K human whole genome oligonucleotide-based arrays, Agilent Technologies) were performed and data were extracted as previously described [15, 16] (compare supplementary materials).

### Cell culture and tissues

Immortalized GBM cell lines were purchased from American Type Culture Collection (ATCC, Manassas, VA, USA) and kept in their respective media (Sigma-Aldrich, St. Louis, MO, USA). Primo-cell cultures were established and RNA extracted from *isocitrate dehydrogenase (IDH)* wild-type GBM surgical specimens in our laboratories (Medical University Vienna, Kepler University Hospital Linz) as described [17, 18]. NCH644 and NCH421K glioma stem cell-like (GSC) models were derived from CLS Cell Lines Service GmbH (Eppelheim, Germany: MTA to FE). Cell culture conditions are outlined in the supplementary materials.

### Protein expression analyses

Western blotting and immunohistochemistry (IHC) were performed as described previously [18] and in the supplementary materials. For Western blotting, the hepatocellular carcinoma model Hep3B, exhibiting high FGFR4 levels [19], was used as positive control. Additional File 3: Table S1 lists all used antibodies and working concentrations. β-actin served as loading control.

### Generation of viral constructs and transduction

Initial plasmids encoding wild-type *FGFR4-388Gly* were kindly provided by Prof. S. Ezzat, M.D. (University Health Network, University of Toronto, Toronto, Ontario, Canada). An *FGFR4 kinase domain-mutated (K504M)* vector encoding an inactivated *FGFR4-kinase dead (KD)* gene was generated by site-directed mutagenesis [20]. For transient *FGFR4* inactivation, cells were incubated with adenoviruses encoding for a CFP-tagged truncated FGFR4 (tFGFR4) [21]. Transduction success and receptor localization were analyzed by flow cytometry, confocal microscopy, Western blots, and qRT-PCR. All details are given in the supplementary materials section.

### qRT-PCR

4 × 10^5^ cells were seeded into 6-well plates. After 24 h, RNA was isolated, reverse transcription performed, and qRT-PCR run [18]. For SYBR-PCR, *RPL-41* served as housekeeping gene. *FGFR4* mRNA levels were measured using Taqman probes (Thermo Fisher Scientific) with FAM/ROX qPCR Mastermix (Thermo Fisher Scientific), and *ACTB* served as housekeeping gene. Hep3B [19] was used as positive control in screening approaches. All RNAs were isolated three times. Additional File 4: Table S2 lists primers and Taqman probes.

### Annexin / PI staining

*tFGFR4-* and *GFP*-transduced GBM cells were stained with Annexin V and PI and measured by flow cytometry (BD LSR Fortessa X-20 Flow Cytometer) as described in the supplementary materials.

### Clonogenicity and proliferation assays

Clonogenicity was analyzed as discussed previously [18]. *tFGFR4* or *GFP* adenoviruses were added one day after seeding and cells were incubated for seven days. Plates were photographed, pictures binarized, and black pixels counted by *R* scripting. To test proliferation, 3 × 10^4^ cells/ml were seeded in 500 µl in 24-well plates. Cells were trypsinized and counted using CASY® cell counter. All experiments were performed at least three times in duplicates.

### Migration assays

Filter-migration assays were performed by Boyden-chambers via a nutrient gradient and cells were incubated for 48 h. For scratch assays, cells were seeded as monolayers and wound-healing capacity was followed, as described in the supplementary materials.

### Adhesion and invasion assays

Adhesion assays towards several coatings were performed in a time-dependent manner. Integrin-mediated cell adhesion was tested according to manufacturer’s manuals (ECM532: Merck, Darmstadt, Germany). A monolayer of m-cherry-tagged endothelial cells was used to test tumor cells´ trans-endothelial invasion. Details are described in the supplementary materials.

### Re-differentiation assay

GBM neurospheres were re-plated in serum-supplemented medium and differentiation plasticity was tested after five days. For details see the supplementary materials.

### Cell-viability assay (MTT)

Ponatinib, BLU554, cilengitide, and defactinib were purchased from Selleck Chemicals (Houston, TX, USA). MTT assays (EZ4U, Biomedica, Vienna, Austria) were performed as previously published [18]. All experiments were performed at least three times. Combination index (CI) values were calculated [22] using CalcuSyn software. CI values < 0.9 were considered synergistic, 0.9–1.2 additive, and > 1.2 antagonistic.

### Xenograft formation experiments

CB-17 severe combined immune-deficient (SCID) mice were subcutaneously injected with 1 × 10^6^ GBM cells. NOD-scid IL2Rgnull (NSG) mice were orthotopically implanted with 5 × 10^5^ tumor cells. The tumor growth and wellbeing of the animals was followed over-time as described in the supplementary materials section. For zebrafish xenografts, see the supplementary materials section. All subcutaneous animal experiments were controlled by the Ethics Committee for the Care and Use of Laboratory Animals at the Medical University Vienna (proposal numbers: BMWF-66.009/0157-II/10b/2008, BMWF-66.009/0157-V/3b/2019). Orthotopic animal experiments were performed in an AAALAC accredited animal facility under the permit G18/78 of the Regierungspräsidium Freiburg im Breisgau, Germany.

### Statistics

In silico analyses were performed and visualized in *R* statistical environment (v.4.0.0) by application of the according packages as described in the supplementary materials. GraphPad Prism 8.0.1 (GraphPad Software, La Jolla, CA, USA) software was used to analyze and visualize raw data. p-values of less than 0.05 were considered statistically significant.

## Results

### FGFR4 overexpression is associated with shorter GBM patient survival and tumor recurrence

**Fig. 1:**
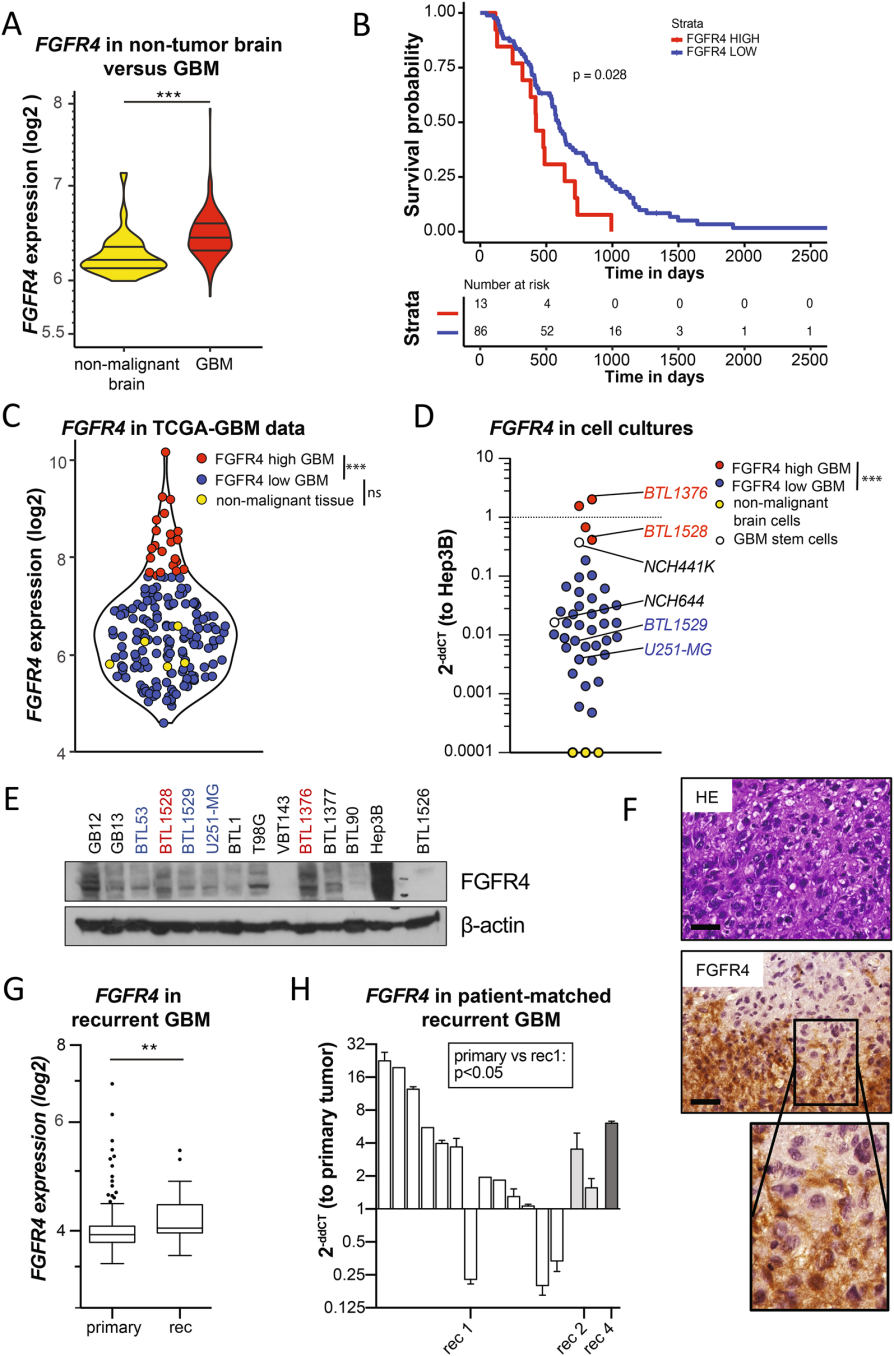
FGFR4 is overexpressed in a highly aggressive GBM subgroup, associated with tumor recurrence. **A** *FGFR4* mRNA levels in non-tumor brain (n = 28) and GBM (n = 219) in the REMBRANDT dataset are shown as violin plots. **B** Kaplan Meier survival analysis of GBM patients from the REMBRANDT dataset stratified for *FGFR4* expression (n = 99.)** C** *FGFR4* mRNA levels in non-malignant brain (yellow, n = 5), low- (blue, n = 147), and high- (red, n = 22) expressing GBM subgroups of the TCGA-GBM RNA sequencing data. **D** *FGFR4* mRNA levels detected by qRT-PCR in GBM (n = 40), glioblastoma stem cells (n = 2, white) models, and non-malignant brain tissue extracts (n = 3, yellow) are shown relatively to Hep3B positive control (2^−ddCT^). *FGFR4*^*high*^ and *FGFR4*^*low*^ GBM models selected for further analyses are highlighted, respectively. **E** Detection of FGFR4 (several bands due to increasing glycosylation) in membrane-enriched fractions from selected GBM cell models and Hep3B by Western blot, with β-actin as loading control. **F** FGFR4 IHC staining of BTL1376 tumor material is opposed to haematoxilin eosin (HE) stain. Scale bars: 50 µm. **G** *FGFR4* mRNA data (TCGA-GBM-HG-U133A) of primary (n = 497) and recurrent (n = 16) GBM are visualized. **H** *FGFR4* mRNA levels in patient-matched primary GBM (n = 14) and sequential recurrences (rec1, n = 13; rec2, n = 2; rec4, n = 1). Statistical analyses: log-rank test **B**; Wilcoxon test **C**; maximization of the t-statistics **D**; Student’s t-tests **A,G,H**;. n.s. = not significant,  *p < 0.05, **p < 0.01, ***p < 0.001

Analyses of several publicly available datasets revealed the brain as an organ with relatively low *FGFR4* expression (Additional File 1: Figure S1A) as compared to other FGFR family members (Additional File 1: Figure S1B), with the highest expression level in the cerebellum (Additional File 1: Figure S1A-C). Concerning GBM, elevated *FGFR4* expression levels were identified in malignant tissue as compared to non-malignant brain (Fig. 1A). Unsupervised sample stratification by maximally selected rank statistics resolved a distinct GBM patient population (13%) with high *FGFR4* expression that was associated with worse prognosis in two independent cohorts (Fig. 1B, Additional File 1: Fig. S2). *FGFR4* showed a broad expression range in the TCGA-GBM cohort (Fig. 1C), which was well reflected in cell cultures on mRNA (Fig. 1D) as well as on protein levels (Fig. 1E). Glioblastoma stem cell (GSC) models exhibited comparably high *FGFR4* mRNA levels (Fig. 1D). Again an *FGFR4*^*high*^ subset was resolved by maximization of the t-statistics (Fig. 1C) and confirmed in corresponding tumor tissues (shown for the BTL1376 FFPE sample in Fig. 1F). Based on the FGFR4 expression screening in GBM, we selected the immortalized cell line U251-MG as well as the primo-models BTL1529 and BTL53 as endogenously *FGFR4*^*low*^, and BTL1528 and BTL1376 as *FGFR4*^*high*^ models for further analyses. High *FGFR4* expression in BTL1528 and BTL1376 was not attributed to gene amplification (Additional File 1: Figure S3A). Concerning GBM progression, *FGFR4* mRNA expression was significantly enhanced in recurrent compared to primary lesions (Fig. 1G, TCGA collection). Analyses of patient-matched primary and recurrent GBM tissues from our clinics corroborated this finding in the majority of cases (Fig. 1H). At this progressed disease stage, expression of FGFR4-specific ligand-coding genes *FGF19* and *FGF23* was distinctly enhanced in the TCGA cohort (Additional File 1: Figure S3B), and our own sample collection (Additional File 1: Figure S3C). Simultaneous upregulation of ligands and receptors is exemplified in a selected GBM patient in Additional File 1: Figure S3D.

### FGFR4 overexpression promotes GBM cell aggressiveness

**Fig. 2:**
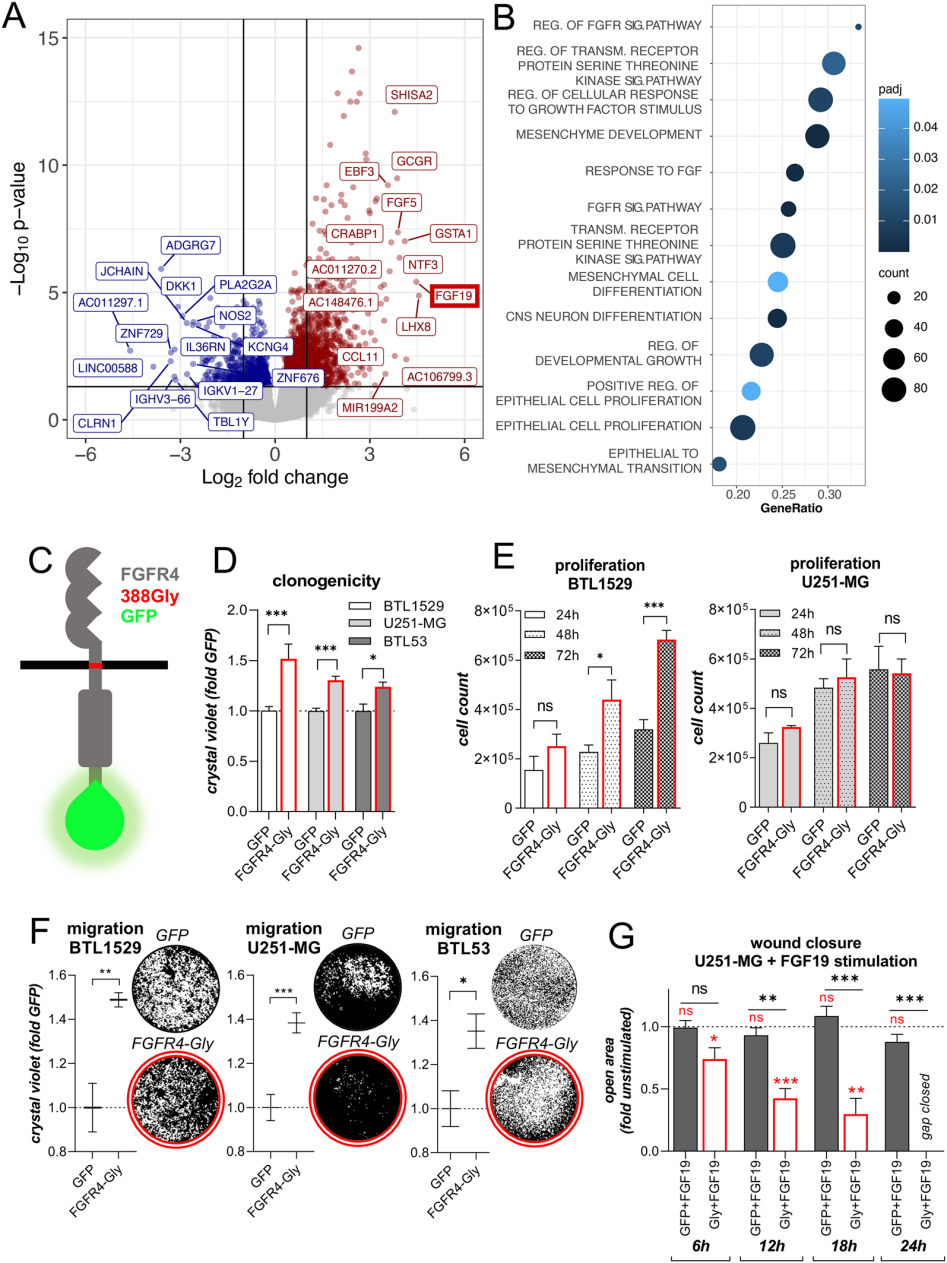
*FGFR4* overexpression promotes GBM aggressiveness. **A** Volcano blot showing differentially expressed genes (DEGs) of TCGA-GBM RNA sequencing data in *FGFR4*^*high*^ (red) versus *FGFR4*^*low*^ (blue) GBM. Top 15 genes are annotated and *FGF19* is highlighted. Adjusted p-value < 0.05. **B** Gene sets significantly enriched in the *FGFR4*^*high*^ GBM subgroup are indicated. GSEA of DEGs **(**in **A)** were performed. Selected gene ontologies are plotted. **C** Scheme of the FGFR4-388Gly-GFP protein. **(D + E)** Clonogenicity **D** and proliferation capacity **E** of the *FGFR4-388Gly*-overexpressing and *GFP-*transduced, endogenously *FGFR4*^*low*^ GBM models are shown (means ± SEM from three independent experiments). **F** Filter-migration capacities of *FGFR4-388Gly*-overexpressing normalized to *GFP*-transduced endogenously *FGFR4*^*low*^ GBM models are shown (mean ± SEM from three experiments). Representative photographs are shown. **G** Wound-healing capacity of *FGFR4-388Gly-*overexpressing and *GFP*-transduced U251-MG cells in response to FGF19 stimulation (50 ng/ml) is shown at the indicated time points. Results were normalized to the respective FGF19-unstimulated conditions (means ± SD from three experiments). Red asterisks: Significance FGF19-stimulated versus -unstimulated. Statistical analyses: 2-way ANOVA/Bonferroni correction in **D,E,G;** Student ‘s t-tests in **(F)**. *p < 0.05, **p < 0.01, ***p < 0.001, ns = not significant

In order to evaluate the impact of FGFR4 on the malignant phenotype of GBM, we performed DESeq2 analyses of the *FGFR4*^*high*^ versus *FGFR4*^*low*^ subgroups of the RNA sequencing TCGA-GBM dataset (compare Fig. 1C). Accordingly, *FGF19* was among the top-ranked genes in the *FGFR4*^*high*^ versus *FGFR4*^*low*^ GBM subset (Fig. 2A). Despite a generally higher expression of *FGF1* and *FGF2* as compared to *FGF19*, the *FGFR4*^*high*^ GBM subgroup expressed reduced *FGF1* and *FGF2,* but enhanced *FGF19* levels (*p* = *E-9*) (Fig. 2A, Additional File 1: Figure S4A). Resembling FGFR4, also expression of *FGF19* was very low in non-malignant brain (Additional File 1: Figure S4B). To test the functional relevance of FGFR4 in our endogenously high- compared to low-expressing GBM models, we stimulated BTL1528 and BTL1529 cells, respectively, with the FGFR4-specific activating ligand FGF19. We observed significantly enhanced clonogenicity and wound-closure potential upon FGF19 stimulation in the *FGFR4*^*high*^ model, while the *FGFR4*^*low*^ model remained unaffected (Additional File 1: Figure S4C-D). Interestingly, FGF19 stimulated sphere formation capacity in both cell models, pointing towards a critical role of FGFR4 signaling in stemness and 3-dimensional growth (Additional File 1: Figure S4E). Together, these data suggest that FGFR4 exhibits a pivotal functional impact on GBM aggressiveness, and that the FGF19/FGFR4 interaction should dominate the FGFR-related signaling in this *FGFR4*^*high*^ GBM subgroup. Using gene set enrichment analyses (GSEA) we found, besides various FGF- and FGFR-related ontologies, growth and differentiation processes including *mesenchyme development* (NES = 1.632), *mesenchymal cell differentiation* (NES = 1.454), *central nervous system neuron differentiation* (NES = 1.709) and *epithelial cell proliferation* (NES = 1.424) significantly associated with high *FGFR4* expression (Fig. 2B and Additional File 5: Table S3). To dissect the underlying cell biological mechanisms in an isogenic background, we overexpressed *wild-type* GFP-tagged *FGFR4-388Gly (FGFR4-388Gly-GFP,* Fig. 2C) in the endogenously *FGFR4*^*low*^ GBM patient-derived cell models BTL1529 and BTL53, and the stable cell line U251-MG. In parallel, GBM models were transduced with a GFP-tagged, dominant-negative FGFR4 version (loss-of-function point mutation *K504M; FGFR4-KD*). Stable mRNA and protein expression as well as FGFR4 functional activity and intracellular localization were confirmed in all GBM sublines (Additional File 1: Figure S5A-D). The effect of empty- and GFP-vector transduction on cell proliferation was negligible (Additional File 1: Figure S6A). Overexpression of *wild-type FGFR4-388Gly* resulted in significantly increased clonogenicity of all GBM models (Fig. 2D) and promoted proliferation capacity of the low-passage primo-cell cultures (Fig. 2E, left; Additional File 1: Figure S6B) but not the high-passage GBM cell line (Fig. 2E, right). In contrast, the migratory potential of all *FGFR4-388Gly-*expressing cells was distinctly enhanced in Boyden chamber (Fig. 2F) and wound-healing assays (Additional File 1: Figure S6C). Stimulation with FGF19 significantly promoted wound closure selectively in *FGFR4-388Gly* GBM cells, exemplarily shown for U251-MG (Fig. 2G). Together, this suggests that FGFR4 exhibits a pronounced growth- and migration-promoting function. This effect is more comprehensively reflected in primary patient-derived cell explants as compared to high-passage GBM cell lines.

### Inactivation of FGFR4 attenuates GBM cell aggressiveness

**Fig. 3:**
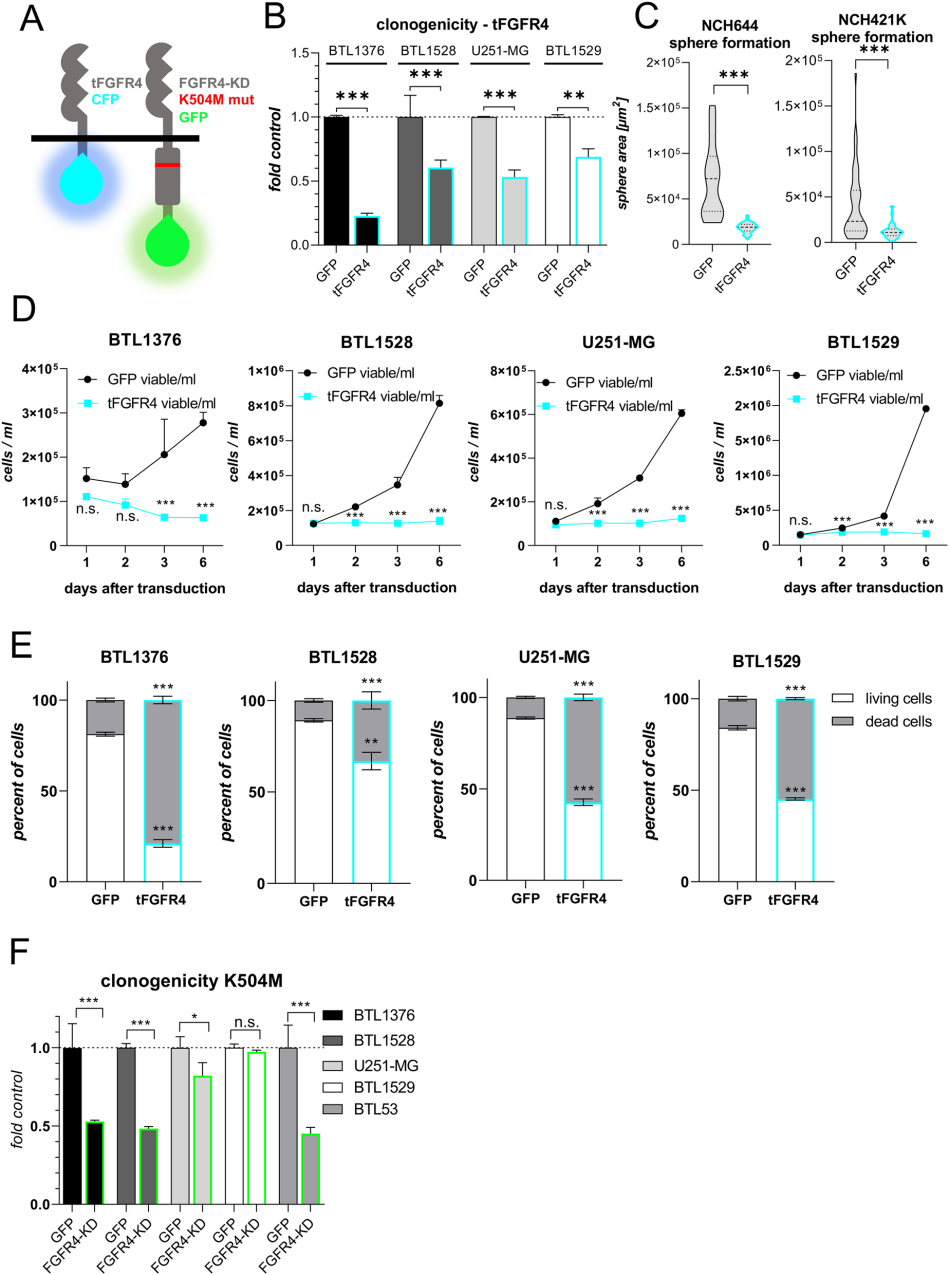
Inactivation of FGFR4 reduces proliferative capacity and promotes cell death. **A** Schemes of the kinase domain-truncated, CFP-coupled *tFGFR4 (left)* and of the point-mutated (mut), *kinase-dead FGFR4-KD(K504M) (right),* GFP-coupled proteins are shown. **(B + F)** Clonogenicity upon *tFGFR4* **B**, *FGFR4-KD(K504M)* **(F)** compared to *GFP* **(B + F,** set to 1**)** transduction was analyzed in endogenously *FGFR4*^*high*^ (BTL1376, BTL1528) or *FGFR4*^*low*^ (U251-MG, BTL1529, BTL53) cell models (mean ± SD from three experiments). **C** Sphere formation potential of GSC models upon *tFGFR4* or *GFP* transduction (mean ± 25%CI). **D** Survival/proliferation capacities over time are shown for *tFGFR4*- and *GFP*-transduced GBM models. For each model, one representative out of three experiments in duplicates is depicted. **E** Cell death induction upon *tFGFR4* or *GFP* transduction in GBM models after 3 days. Annexin/PI staining was analyzed by flow cytometry. Significance levels were calculated comparing *tFGFR4-* to *GFP-*transduced cells on the respective living or dead fraction. Statistical analyses: 2-way ANOVA/Bonferroni correction (mean ± SD) **(B,D,E,F);** Student’s t-tests **(C)**. *p < 0.05, **p < 0.01, ***p < 0.001, n.s. = not significant

The consequences of FGFR4 inactivation in GBM were assessed by two approaches (Fig. 3A). First, the above described *FGFR4-KD-GFP(K504M)* vector was transduced generating stable FGFR4-inactivated GBM models (compare Additional File 1: Figure S5). Second, for transient FGFR4 blockade, an adenoviral construct encoding a truncated *FGFR4* gene variant *(tFGFR4)* with the intracellular kinase domain exchanged by CFP was applied (Fig. 3A, Additional File 1: Figure S7A). *tFGFR4* transduction into all tested glioma cells led to significantly impaired clone formation (Fig. 3B) and proliferation potential (Fig. 3D) of GBM cell models. Concerning stemness, *tFGFR4* significantly reduced sphere formation capacity in GSC models (compare Fig. 3C). In parallel, the spontaneous cell death rate in several GBM models increased upon *tFGFR4* infection (Additional File 1: Figure S7A-B and Fig. 3E). Accordingly, introduction of *FGFR4-KD(K504M)* impaired GBM clonogenicity and cell proliferation, especially in the endogenously *FGFR4*^*high*^ glioma models (Fig. 3F, Additional File 1: Figure S7C, respectively). This effect was less pronounced as compared to *tFGFR4* infection (compare Fig. 3B), possibly due to cellular compensatory mechanisms upon FGFR4 inactivation in the stable *FGFR4-KD(K504M)*-overexpressing cell models. Accordingly, genes encoding for *FGFR2*, *FGFR3* and the universal FGFR-activating ligand *FGF1* were upregulated in response to *FGFR4-KD(K504M)* expression (Additional File 6: Table S4).

### FGFR4 inactivation attenuates GBM cell migration and endothelial barrier disintegration

**Fig. 4:**
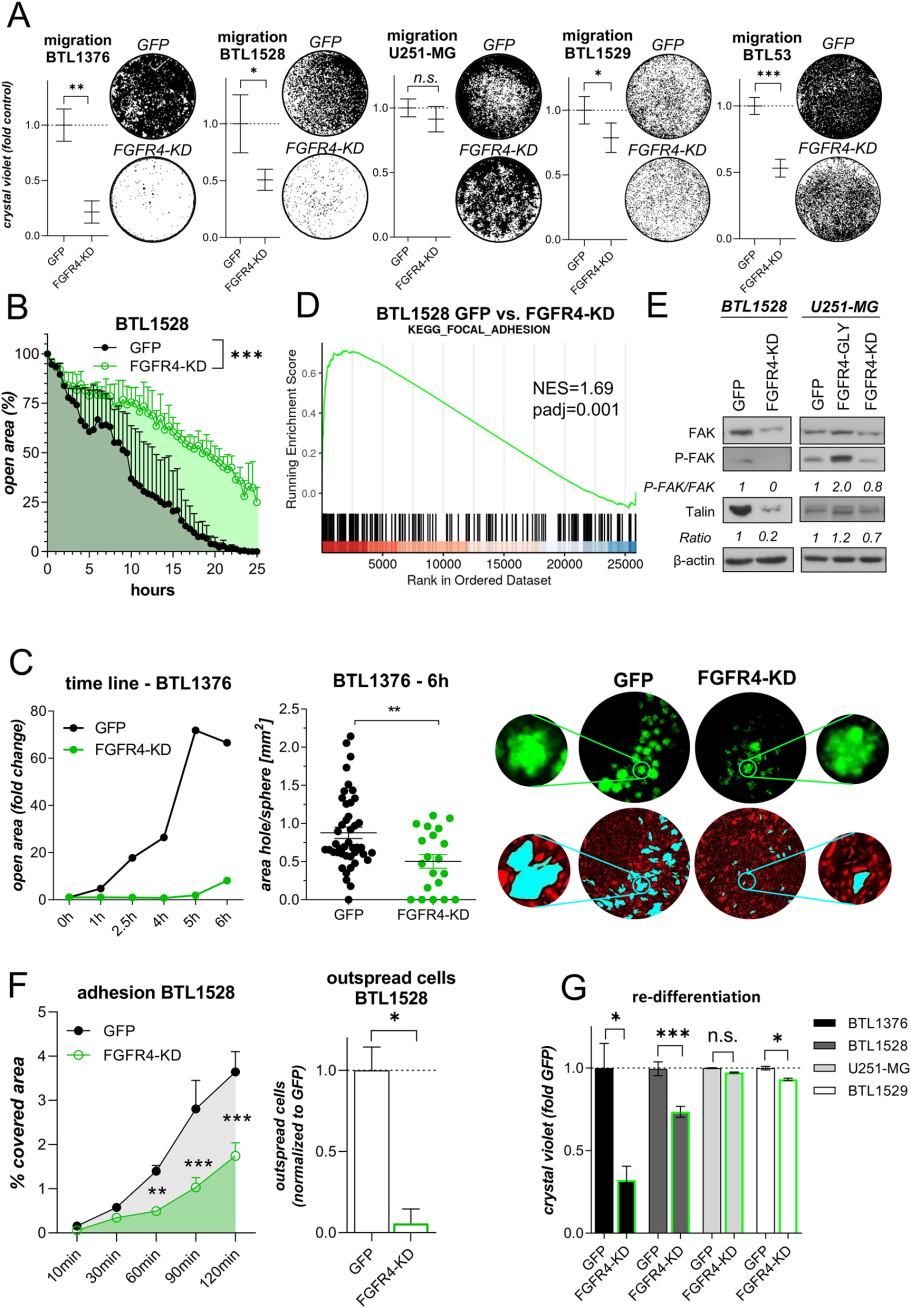
FGFR4 inactivation attenuates GBM cell migration, endothelial invasion, and adhesion. **A** Filter-migration of *FGFR4-KD(K504M)-* and *GFP-*transduced GBM sublines is shown (mean ± SEM from three experiments). Representative photographs are depicted. **B** Wound-healing capacities of BTL1528 *FGFR4-KD(K504M)-* and *GFP-*transduced cells were evaluated (means ± SEM of one representative experiment in triplicates). **C** Trans-endothelial invasion capacity of *FGFR4-KD(K504M)-* and *GFP-*transduced BTL1376 neurospheres into m-cherry-tagged blood endothelial cells (BEC) was evaluated by live-cell microscopy *(left).* Generated “wounds” after 6 h co-culture were normalized to the respective sphere sizes *(middle*)*.* Representative photomicrographs of tumor spheres (GFP), BEC (m-cherry) and invasion wounds (highlighted in turquoise) are depicted *(right).* **D** *KEGG_FOCAL_ADHESION* gene set from GSEA analyses of BTL1528 *GFP* versus *FGFR4-KD(K504M)* cells is shown. **E** FAK expression and phosphorylation and talin expression in the indicated *FGFR4*-modulated GBM sublines compared to GFP-transduced controls was detected by Western blotting with β-actin as loading control. Ratios were calculated by normalization to β-actin and are shown as fold change to GFP-transduced cells. **F** Adhesion capacities of BTL1528 *FGFR4-KD(K504M)* and *GFP* control cells as percentage of well surfaces covered with cells (means ± SEM of three experiments) *(left).* Outspread cells (120 min) were counted microscopically (normalized to GFP control cells) *(right).* **G** Re-differentiation capacity of the indicated GBM spheroids from *FGFR4-KD(K504M)* cells normalized to the respective *GFP* controls are shown (mean ± SEM from three experiments). Statistical analyses: Student’s t-tests **(A)**,**(C middle; F right)**; area under the ROC curve analysis **(B)**; 2-way ANOVA/Bonferroni correction **(F left, G)**. *p < 0.05, **p < 0.01, ***p < 0.001, n.s. = not significant; NES = normalized enrichment score, padj = adjusted *p*-value

Overexpression of *FGFR4-KD(K504M)* significantly reduced migration capacity in all tested primo-GBM models, but not in the stable cell line U251-MG (Fig. 4A). Furthermore, the wound-healing capacity was significantly impaired upon FGFR4 inactivation as representatively shown for BTL1528 (Fig. 4B). For in vitro investigation of GBM cell invasiveness, spheres of BTL1376 *FGFR4-KD(K504M)* or *GFP* control cells were transferred onto a monolayer of blood endothelial cells. The endothelial cell layer invasion capacity of *FGFR4*^*high*^ GBM cells was drastically impaired upon FGFR4 inactivation (Fig. 4C). To dissect the underlying mechanisms, we performed GSEA of the *FGFR4-KD(K504M)* subclones of both endogenously *FGFR4*^*high*^ GBM models. The *KEGG Focal Adhesion* pathway appeared amongst the highest enriched ontologies in BTL1528 as well as in BTL1376 (Fig. 4D and Additional File 7: Table S5, Additional File 1: Figure S8A and Additional File 8: Table S6, respectively). This translated well into regulated focal adhesion kinase (FAK) expression and phosphorylation levels, as well as talin protein expression in response to FGFR4 manipulation (Fig. 4E, Additional File 1: Figure S8B), resulting in significantly diminished adhesion potential to cell culture polystyrene in several GBM (Fig. 4F, Additional File 1: Figure S8C + D, left) and GSC (Additional File 1: Figure S8E) models upon FGFR4 blockade. Corroboratively, the re-differentiation capacity was impaired by *FGFR4-KD(K504M)*, especially in endogenously *FGFR4*^*high*^ GBM models (Fig. 4G). Regardless of endogenous FGFR4 expression levels, *tFGFR4* transduction significantly reduced the re-differentiation capacity of all tested GBM (Additional File 1: Figure S8F) and GSC (Additional File 1: Figure S8G) cultures. Furthermore, pharmacological FGFR4 inhibition by either BLU554 or ponatinib distinctly reduced expression of the stemness marker nestin (Additional File 1: Figure S8H), correlating with significantly impaired all-trans retinoic acid (ATRA)-induced differentiation of NCH644 GSC (Additional File 1: Figure S8I).

### FGFR4 regulates integrin-mediated GBM cell adhesion

**Fig. 5:**
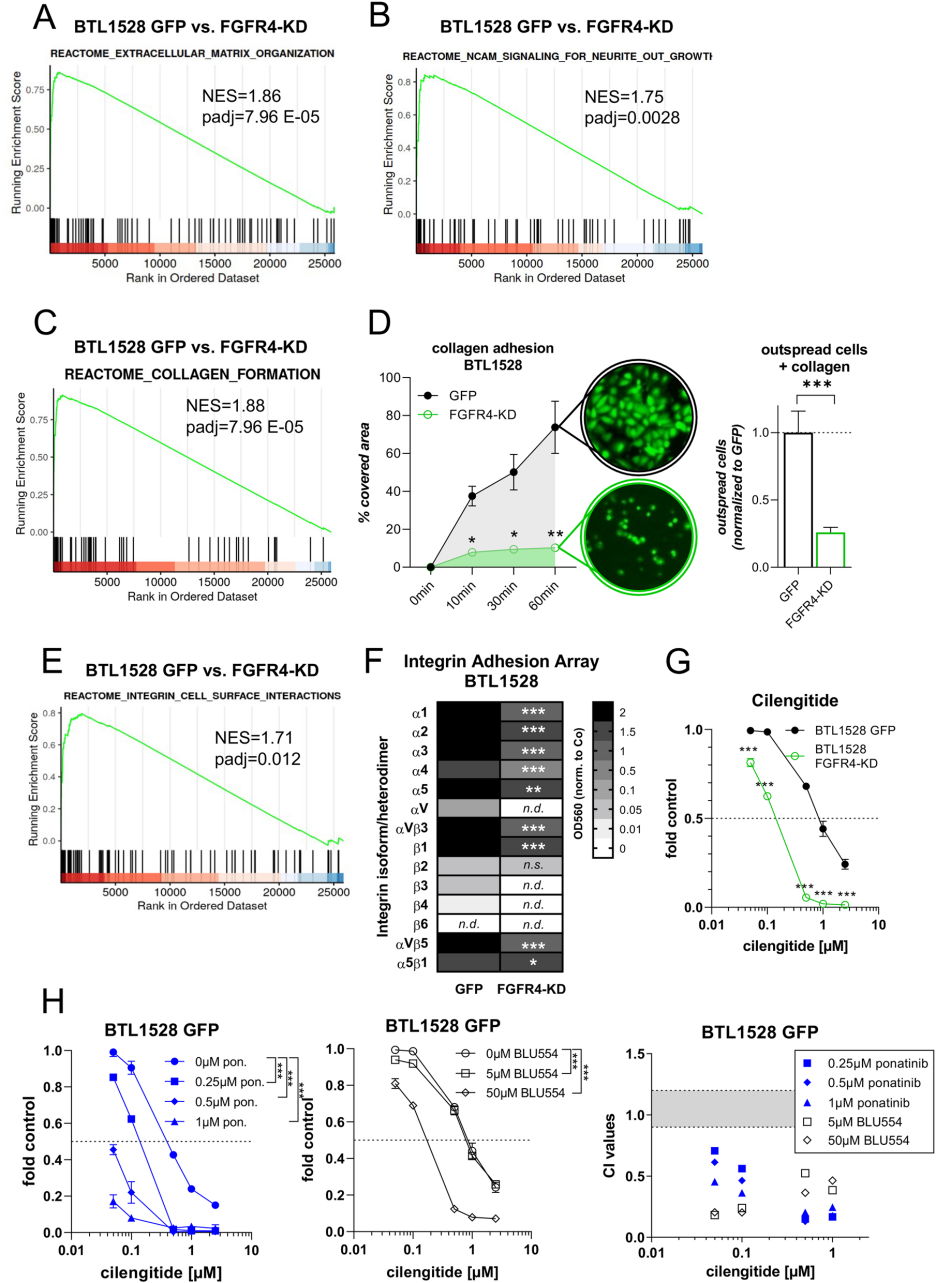
FGFR4 blockade results in loss of GBM cell adhesion and sensitizes towards the *RGD*-mimetic cilengitide. **A-C,E** *REACTOME* gene sets enriched in BTL1528 *GFP* versus *FGFR4-KD(K504M)* gene expression data based on GSEA. NES = normalized enrichment score, padj = adjusted p-value. **D** Adhesion capacities of BTL1528 *FGFR4-KD(K504M)* and respective *GFP* cells towards collagen as percentage of cell-coated well surfaces (means ± SEM of three experiments *(left)*. Exemplary photomicrographs are shown. Outspread cells after 60 min were counted microscopically and data normalized to *GFP* control cells *(right).* **F** Integrin-mediated cell adhesion arrays of *FGFR4-KD(K504M)-* and *GFP-*transduced BTL1528 subclones are shown (means of two experiments). **(G + H)** Viability of BTL1528 *FGFR4-KD(K504M)-* and *GFP-*expressing GBM cells in response to single-agent cilengitide **(G),** combined-agent cilengitide + ponatinib (pon.) **(H left)** and cilengitide + BLU554 **(H middle)** was evaluated by MTT assays. For each panel, one representative out of three experiments is shown as mean ± SD from triplicates. Combination indices (CI) are given for selected drug concentrations combining cilengitide with ponatinib (blue) or BLU554 (black) **(H right)**. CI values < 0.9 were considered synergistic [22]*.* Statistical analyses: 2-way ANOVA/Bonferroni correction **(D left, F–H)**, Student’s t-tests **(D right)**. *p < 0.05, **p < 0.01, ***p < 0.001, n.s. = not significant, n.d. = not detected

Unsupervised GSEA of both tested GBM models, BTL1528 (Fig. 5A–C, Additional File 1: Figure S10A, Additional File 7: Table S5) and BTL1376 (Additional File 1: Figure S9A–C, Additional File 8: Table S6), further revealed alterations in REACTOME pathways including *extracellular matrix organization*, *NCAM signaling for neurite outgrowth,* and *collagen formation* following FGFR4 inactivation. By applying identical GSEA settings to the more comprehensive TCGA-GBM RNA sequencing dataset, all three above listed pathways were ranked as the most significantly enriched REACTOME processes in the *FGFR4*^*high*^ versus *FGFR4*^*low*^ subgroup (padj = 0.002, NES = 1.79; padj = 0.0004, NES = 1.92; padj = 0.0004, NES = 1.92, respectively; Additional File 5: Table S3). Enrichment network analyses of BTL1528 subclones identified these gene sets clustering together in one functional module (Additional File 1: Figure S10B, violet module). Accordingly, collagen coating distinctly promoted FGFR4-dependent GBM cell adhesion (Fig. 5D compare Fig. 4F, middle panels of Additional File 1: Figure S8C + D). In addition to decreased expression of collagen network genes upon FGFR4 inactivation (compare Fig. 5C and Additional File 1: Figure S9C), we also found loss of *integrin-cell surface interactions*-related genes in both GBM models (BTL1528: Fig. 5E and BTL1376: Additional File 1: Figure S9D). This translated well into consistently downregulated integrin-mediated cell adhesion via various integrin isoforms and heterodimers in *FGFR4-KD(K504M)*-overexpressing *FGFR4*^*high*^ BTL1528 cells (Fig. 5F). Accordingly, expression of several integrin members including integrin αV was changed following FGFR4 manipulation (Additional File 1: Figure S9E + F). A functional impact of the diminished integrin αV protein expression was also suggested by reduced adherence capacity towards fibronectin (Additional File 1: Figure S8C + D right, S9G). Strikingly, *FGFR4-KD(K504M)* expression led to distinct hypersensitivity towards the *Arg-Gly-Asp (RGD)* motif-specific integrin inhibitor cilengitide in both GBM models (Fig. 5G, Additional File 1: Fig. S9H). Also the FAK inhibitor defactinib, targeting focal adhesion-related integrin downstream signaling, was hyperactive in *FGFR4-KD(K504M)* subclones (Additional File 1: Figure S9I + J), suggesting cooperation between FGFR4 and integrin-mediated signals promoting GBM cell survival. This hypothesis was supported by synergistic actions of pharmacological FGFR4 inhibition with both the multi-FGFR inhibitor ponatinib or the FGFR4-specific tyrosine kinase inhibitor (TKI) BLU554 with cilengitide in the endogenously *FGFR4*^*high*^ model BTL1528 (Fig. 5H).

### FGFR4 promotes GBM tumorigenicity

**Fig. 6:**
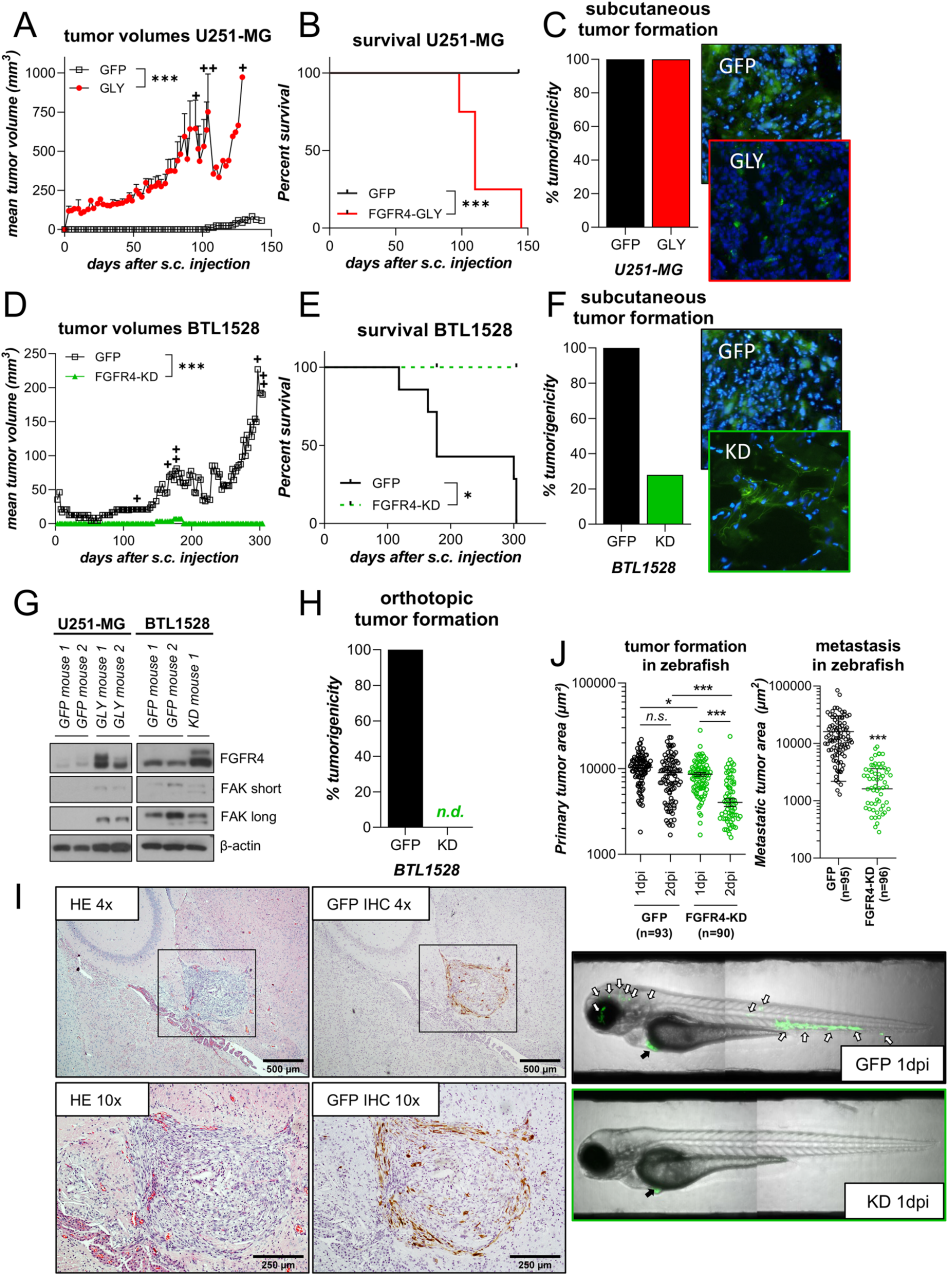
FGFR4 impacts on tumorigenicity and tumor growth. **A-F** SCID/CB17 mice were subcutaneously injected with wild-type *FGFR4-388Gly-*expressing, endogenously *FGFR4*^*low*^ U251-MG cells or *GFP*-transduced controls (n = 4/group) **(A-C)** or kinase-dead *FGFR4-KD(K504M)-*expressing, endogenously *FGFR4*^*high*^ BTL1528 cells or *GFP*-transduced controls (n = 7/group) **D-F**. Tumor growth curves (mean tumor volume ± SEM). +  = mouse sacrificed **(A/D)**, Kaplan Meier survival curves **(B/E)**, tumor take **(C/F, left)**, and representative cryo-sections of DAPI stained tumor xenografts **(C/F, right)** are shown. **G** Western blot analysis of the indicated proteins in U251-MG (*left*) or BTL1528 (*right*) *FGFR4*-altered xenografts (compare panels A-F). β-actin served as loading control. **H** Orthotopic tumor formation of BTL1528 *GFP* and *FGFR4*-modulated sublines in mice (n = 5/group) is shown. **(I)** Representative photomicrographs of a BTL1528 *GFP* orthotopic tumor stained with HE (*left panels*) or GFP by IHC (*right panels*). **J** Primary tumor area over time (*left*) and extra-primary tumor cell migration in zebrafish larvae (*right*) of BTL1528 *GFP-* or *FGFR4-KD(K504M)*-expressing models one-day post injection (dpi). Data from the independent experiments are shown. Representative photomicrographs of zebrafish larvae are depicted in the lower panels. black arrows: primary tumors, white arrows: extra-primary site tumor cell clusters; 2-way ANOVA **(A + D; J left)**, log-rank (Mantel-Cox) test **(B + E)**, or Student’s t-test **(J right)**. n.d. = no detectable tumors, n.s. = not significant, *p < 0.05, ***p < 0.001. *GLY* = *FGFR4-388Gly, KD* = *FGFR4-KD(K504M),* s.c. = subcutaneous, short/long: exposure times

One endogenously *FGFR4*^*low*^ (U251-MG) and one *FGFR4*^*high*^ (BTL1528) GBM cell model as well as their *FGFR4*-altered sublines were next tested for xenograft formation in SCID mice. While both U251-MG sublines were tumorigenic in all animals tested (Fig. 6C), upregulation of wild-type *FGFR4-388Gly* in the endogenously *FGFR4*^*low*^ U251-MG cells resulted in significantly enhanced tumor volumes (Fig. 6A) and shorter overall survival (Fig. 6B) as compared to *GFP-*control tumor-bearing mice. FGFR4 inactivation in the *FGFR4*^*high*^ BTL1528 model resulted in significantly reduced tumor volumes (Fig. 6D) and longer overall survival times of the animals (Fig. 6E). In contrast to the notoriously tumorigenic *GFP* variants, *FGFR4-KD(K504M)* transplants engrafted only in 2/7 mice in BTL1528 (Fig. 6F). Transgene positivity of the xenograft tumors was validated by GFP fluorescence on cryo-sections (Fig. 6C + F, right). The profound impact of FGFR4 inactivation on tumor take might again be related to the above-described impact of FGFR4 on FAK expression, which was confirmed in vivo by immunoblots of xenograft protein extracts (Fig. 6G). The in vivo effects of FGFR4 on BTL1528 aggressiveness were even more distinct at the orthotopic implantation site. Intracranial injection of BTL1528 *GFP* cells led to tumor bulk formation in 100% of cases (n = 5) (Fig. 6H + I), while none of the BTL1528 *FGFR4-KD(K504M)*-implanted mice (n = 5) developed detectable tumors (Fig. 6H). In addition to mouse xenotransplantation models, the impact of FGFR4 on GBM aggressiveness was tested in the zebrafish larvae *(Danio rerio)* model system. Again, *FGFR4*^*high*^* GFP* cell fluorescence widely persisted at the injection site, while BTL1528 *FGFR4-KD(K504M)* tumors significantly regressed after 2 days (Fig. 6J left). Additionally, significantly less cells migrated away from the primary tumor site in case of the FGFR4-blocked BTL1528 model, as compared to its *GFP* subline (Fig. 6J right and photomicrographs), confirming a key role of FGFR4 on the invasive potential of this *FGFR4*^*high*^ GBM model.

## Discussion

Members of the FGF/FGFR signaling network were suggested to regulate several hallmarks of GBM aggressiveness, such as invasion, self-renewal, tumor growth, and therapy resistance [4, 23]. However, a potential role of FGFR4 has not been addressed comprehensively yet. Based on extensive in silico and wet lab analyses of GBM tissues, tumor explants, and GSC models, we have identified FGFR4 mRNA and protein overexpression in a distinct GBM subgroup, which was traced back to the original patient tumor samples by IHC. Based on functional in vitro investigations in stable cell lines and patient-derived explant models, we identified FGFR4 as central mediator of GBM cell aggressiveness, as previously published for melanoma [21]. FGFR4 blockade significantly diminished GBM invasiveness in zebrafish and site-specifically impacted on mouse xenograft formation. In particular, tumorigenicity was completely abolished at orthotopic, but only partially at heterotopic transplantation sites, suggesting specific interactions of FGFR4 with the brain microenvironment.

The question arises, which mechanisms are underlying the overexpression of FGFR4 in this distinct GBM patient subgroup. Alterations of the *FGFR4* gene have only very rarely been detected in GBM tissue [4, 11], and also our *FGFR4*^*high*^ cell models lacked selective amplifications of the respective chromosomal region. Across all FGFR family members, we observed weakest expression levels for *FGFR4* in human non-malignant brain. Solely in the human cerebellum meaningful *FGFR4* and *FGF19* mRNA levels were identified, in line with pig brain data (GTEx portal). However, cerebellar GBM accounts for less than 1% of all cases [24], and no enrichment in our *FGFR4*^*high*^ subgroup was found. In contrast to the adult situation, *FGFR4* expression is present in the embryonic brain and was even suggested as specific marker for neural stem cells in rats [25]. *FGFR4* overexpression is prominent in astroglial cells, promoting astrocyte trans-differentiation towards neural progenitor cells [26]. Interestingly, adult brain neural stem cells are *FGFR4*-negative [27]. In our in silico analyses of the TCGA-GBM cohort, expression of the well-known embryonic stem cell markers *NANOG* and *GLI1* [28] was significantly enhanced in the *FGFR4*^*high*^ subgroup (mRNA log2 fold change (FC): 0.78 and 2.42, respectively). Furthermore, GSC tended to show higher *FGFR4* gene expression, and FGFR4 inhibition significantly reduced GSC differentiation. Together, this presumes reactivation of an embryonic neural stem cell program in *FGFR4*^*high*^ tumors.

Various gene sets related to enhanced cell adhesion and mesenchymal cell differentiation were within the most significantly enriched gene sets in the *FGFR4*^*high*^ subsets of the TCGA-GBM dataset and our own genetically altered cell models. Moreover, one GBM patient treated at our clinic presented with repeated recurrences accompanied by steadily increasing FGFR4 levels and a histological change towards a gliosarcoma. Additionally, the original tumor of the *FGFR4*^*high*^ patient-derived GBM model BTL1376 was histologically classified as gliosarcoma. Thus, we hypothesized a relative enrichment of *FGFR4*^*high*^ cases in the mesenchymal GBM subtype, which has been associated with a particularly high migratory and invasive phenotype [29]. However, although mesenchymal GBM showed significantly higher *FGFR4* levels in the REMBRANDT dataset, we could not validate this finding in the TCGA-GBM dataset. Consequently, the relationship between FGFR4 overexpression and mesenchymal differentiation does not seem straight-forward and needs further in-depth investigations.

The FGF19/FGFR4 axis has been connected to dismal patient prognosis and disease progression in different tumor entities including HCC and breast cancer [5, 12, 23, 30, 31]. Additionally, FGFR4 was identified as key factor inducing proliferation, metastatic disease, and cell dedifferentiation in aggressive luminal A-like breast cancer [31]. Accordingly, we detected shorter overall survival of the *FGFR4*^*high*^ patient subgroups from several datasets, and *FGFR4-KD(K504M)* tumor-bearing mice survived significantly longer as compared to endogenously *FGFR4*^*high*^ control xenografts. Regarding GBM, we found significantly elevated expression of *FGFR4* and associated activating ligand genes in the majority of radio-/chemotherapy-refractory recurrent tumors, based on both in silico and surgical specimen analyses. Consistently, *FGF19* appeared among the highest upregulated genes in the *FGFR4*^*high*^ subgroup. Consequently, these findings further support a role of FGFR4 in GBM recurrence in a highly aggressive GBM subset based on an autocrine loop connecting FGF19 and FGFR4, as previously reported in breast cancer [30].

By introducing activating and dominant-negative *FGFR4* constructs into an isogenic GBM background, a broad impact of FGFR4 on the malignant phenotype of GBM cells in vitro was elucidated. While transient expression of the kinase domain-truncated FGFR4 version efficiently induced cell death, transduction with the point-mutated, kinase-dead FGFR4 variant allowed stable clone selection. This suggests that *FGFR4-KD*(*K504M)-*transduced cells partially bypass the FGFR4 blockade, probably via a so-called receptor tyrosine kinase (RTK)-switch. Indeed, we found other FGFR family members distinctly upregulated upon *FGFR4-KD(K504M)*-mediated blockade. In addition, overexpression of *FGFR4-KD(K504M)*, despite lacking downstream signaling activation, might still deliver kinase-independent, tumor-promoting signals to GBM cells. Interactions of FGFR4 extracellular and transmembrane domains with alternative binding partners such as N-Cadherin or NCAM have been proposed [12]. Furthermore, FGFR4 was reported to form complexes with N-Cadherin and MT1-MMP in the cell membrane, altering cell adhesion properties and facilitating protease-dependent collagen invasion [12].

Consistently, in vitro and in vivo (zebrafish larvae) data demonstrated a supportive role for FGFR4 in GBM cell migration, invasion, and adhesion, which was corroborated by gene expression analyses of our *FGFR4*-engineered glioma cell models and the TCGA-GBM data cohort. The most significantly altered gene ontologies in relation to FGFR4 functionality were consistently associated with cell adhesion and integrin-related extracellular matrix (ECM) interaction mechanisms. This is well in agreement with the massive inhibitory effect of *FGFR4-KD(K504M)* expression on wound-healing capacity*,* reflecting in many aspects the invasive tumor leading edge. Integrins constitute a heterodimeric transmembrane glycoprotein receptor family for ECM components frequently associated with tumor progression. Multiple studies suggested complex formation between different RTKs and integrins, leading to therapy resistance and tumor progression [32, 33]. Integrin αV is part of the so called *RGD-*binding integrin subgroup, a target motif in ECM components like fibronectin and vitronectin [33], and is particularly important in glioma pathogenesis [34]. We found that FGFR4 blockade consistently attenuated integrin αV expression as well as dimerization with various β-isoforms. Functionally, integrin-mediated cell adhesion towards several coatings was distinctly impaired upon FGFR4 inactivation. In addition, clear-cut hypersensitivity of *FGFR4-KD(K504M)* subclones towards two integrin axis-targeting compounds, the integrin antagonist cilengitide and the FAK inhibitor defactinib, was detected. Focal adhesion assembly involving integrin dimers is closely regulated by FAK and talin [33], which were reduced in *FGFR4-KD(K504M)-*transduced GBM cells and in associated xenografts. Pharmacological FGFR inhibition by either the multi-TKI ponatinib or the FGFR4-specific drug BLU554 sensitized *FGFR4*^*high*^ glioma cells towards integrin-targeting. Hence, combined FGFR4 and integrin-/FAK inhibition might be a highly active therapy strategy for the here described aggressive GBM subpopulation. The feasibility of this approach needs to be confirmed in further (pre-)clinical studies.

## Conclusion

Although widely absent in non-malignant brain, here we revealed a pro-tumorigenic function of FGFR4 in GBM. FGFR4 overexpression was identified in a subgroup of GBM patients, predicting shorter survival times. In parallel, the specific receptor-activating ligand FGF19 was coregulated in this patient cohort, suggesting an oncogenic feedback loop mediated by the FGF19-FGFR4 axis. Expression levels of FGFR4 and its specific ligands were significantly enhanced in recurrent diseases. Screening a broad collection of GBM cell models corroborated a distinct FGFR4-high subgroup in vitro*.* By genetic overexpression and knock-down experiments we proved that FGFR4 regulates several classical hallmarks of GBM, including clonogenicity, proliferation, differentiation, migration, and eventually invasiveness by closely interconnecting with the integrin signaling network. FGFR4 blockade significantly reduced tumor growth and progression in subcutaneous murine and in zebrafish xenografts, respectively, and completely diminished tumor formation in mouse brains. Consequently, our data suggest combination approaches targeting both FGFR4 and integrin signaling as novel therapeutic concept in FGFR4-high GBM patients.

## Supplementary Information

**Additional file1. Supplementary Figures**

**Additional file2. Supplementary Materials**

**Additional file3. Supplementary Table 1**

**Additional file4. Supplementary Table 2**

**Additional file5. Supplementary Table 3**

**Additional file6. Supplementary Table 4**

**Additional file7. Supplementary Table 5**

**Additional file8. Supplementary Table 6**

## Data Availability

The REMBRANDT and TCGA-GBM mRNA expression data sets were downloaded from public sources (Gene expression omnibus: GSE108474 and GDC data portal, respectively). mRNA expression data sets from patient-derived GBM cell models will be available from the corresponding author on reasonable request. GSEA data are provided in the supplementary tables.
