# Supplementary material for: Fibroblast growth factor receptor 4 promotes glioblastoma progression: a central role of integrin-mediated cell invasiveness
Source: Acta Neuropathol Commun. 2022 Apr 28;10:65. doi: 10.1186/s40478-022-01363-2 (PMC9052585; doi:10.1186/s40478-022-01363-2)
Supplement: Supplementary file 1 — Additional file1. Supplementary Figures [file 40478_2022_1363_MOESM1_ESM.pdf]

# Figure S1

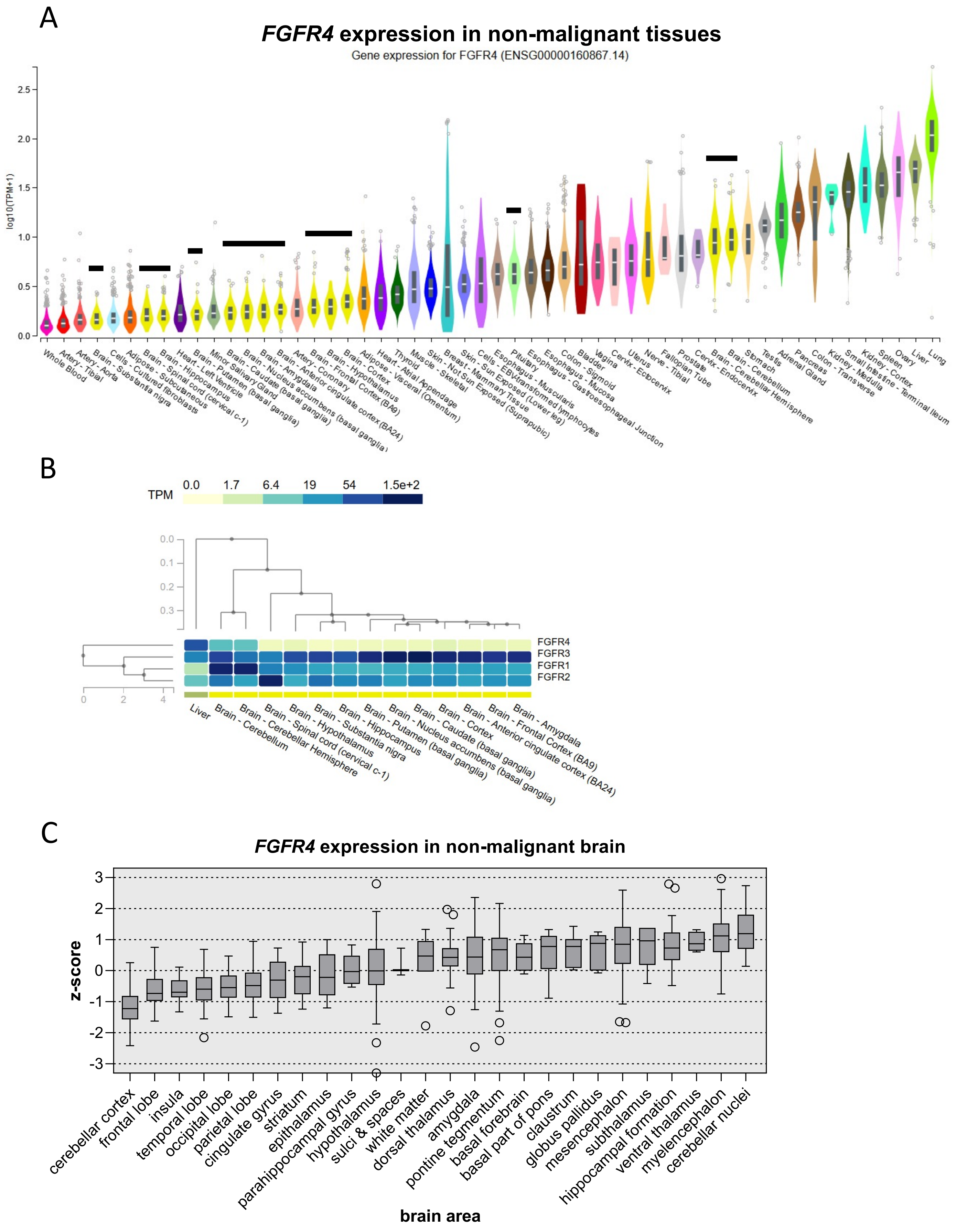
A
FGFR4 expression in non-malignant tissues
Gene expression for FGFR4 (ENSG00000160867.14)
log10(TPM+1)
0.0
0.5
1.0
1.5
2.0
2.5
Whole Blood
Artery - Tibial
Artery - Aorta
Brain - Substantia nigra
Cells - Cultured fibroblasts
Adipose - Subcutaneous
Brain - Spinal cord (cervical c-1)
Brain - Hippocampus
Heart - Left Ventricle
Brain - Putamen (basal ganglia)
Minor Salivary Gland
Brain - Caudate (basal ganglia)
Brain - Nucleus accumbens (basal ganglia)
Brain - Amygdala
Brain - Anterior cingulate cortex (BA24)
Artery - Coronary
Brain - Frontal Cortex (BA9)
Brain - Hypothalamus
Brain - Cortex
Adipose - Visceral (Omentum)
Heart - Atrial Appendage
Thyroid
Muscle - Skeletal
Skin - Not Sun Exposed (Suprapubic)
Breast - Mammary Tissue
Skin - Sun Exposed (Lower leg)
Cells - EBV-transformed lymphocytes
Esophagus - Muscularis
Pituitary
Esophagus - Gastroesophageal Junction
Colon - Sigmoid
Esophagus - Mucosa
Bladder
Vagina
Cervix - Ectocervix
Uterus
Nerve - Tibial
Fallopian Tube
Prostate
Cervix - Endocervix
Brain - Cerebellar Hemisphere
Brain - Cerebellum
Stomach
Testis
Adrenal Gland
Pancreas
Colon - Transverse
Kidney - Medulla
Small Intestine - Terminal Ileum
Kidney - Cortex
Spleen
Ovary
Liver
Lung
B
TPM
0.0
1.7
6.4
19
54
1.5e+2
0.0
0.1
0.2
0.3
0
2
4
FGFR4
FGFR3
FGFR1
FGFR2
Liver
Brain - Cerebellum
Brain - Cerebellar Hemisphere
Brain - Spinal cord (cervical c-1)
Brain - Hypothalamus
Brain - Substantia nigra
Brain - Hippocampus
Brain - Putamen (basal ganglia)
Brain - Nucleus accumbens (basal ganglia)
Brain - Caudate (basal ganglia)
Brain - Cortex
Brain - Anterior cingulate cortex (BA24)
Brain - Frontal Cortex (BA9)
Brain - Amygdala
C
FGFR4 expression in non-malignant brain
z-score
3
2
1
0
-1
-2
-3
cerebellar cortex
frontal lobe
insula
temporal lobe
occipital lobe
parietal lobe
cingulate gyrus
striatum
epithalamus
parahippocampal gyrus
hypothalamus
sulci & spaces
white matter
dorsal thalamus
amygdala
pontine tegmentum
basal forebrain
basal part of pons
claustrum
globus pallidus
mesencephalon
subthalamus
hippocampal formation
ventral thalamus
myelencephalon
cerebellar nuclei
brain area

**Supplementary Figure S1. *FGFR4* expression is comparably low in the brain and differs in between brain regions. (A)** *FGFR4* mRNA expression levels were compared between non-malignant human organs, including different brain regions (indicated by black bars). RNA sequencing data from the Genotype-Tissue Expression (GTEx) Project data set were used. Data are given as log10 transformed transcript per million (TPM) reads. **(B)** Expression levels of the FGFR family members in different brain regions and the liver are shown. Data were obtained from the GTEx project given as TPM. **(C)** *FGFR4* mRNA expression is shown in different adult non-malignant brain areas. Data were obtained from the *Allen brain atlas (brain-map.org)* and are presented as z-scores (box-whiskers: Tukey).

# Figure S2

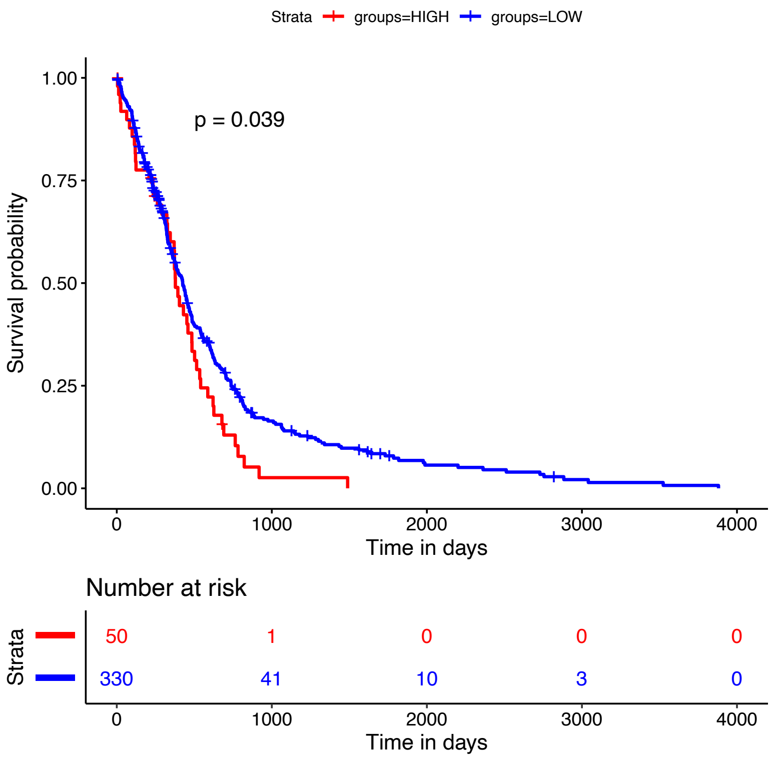


**Supplementary Figure S2. *FGFR4* expression is associated with worse prognosis in GBM patients.** Survival of patients with high (red, n=50) or low (blue, n=330) *FGFR4*-expressing GBM from the TCGA-GBM-395.MAS5.0-u133a data set is shown. Cutpoints for *FGFR4* stratification were determined by maximally selected rank statistics. Statistical analyses: Log-rank tests

# Figure S3

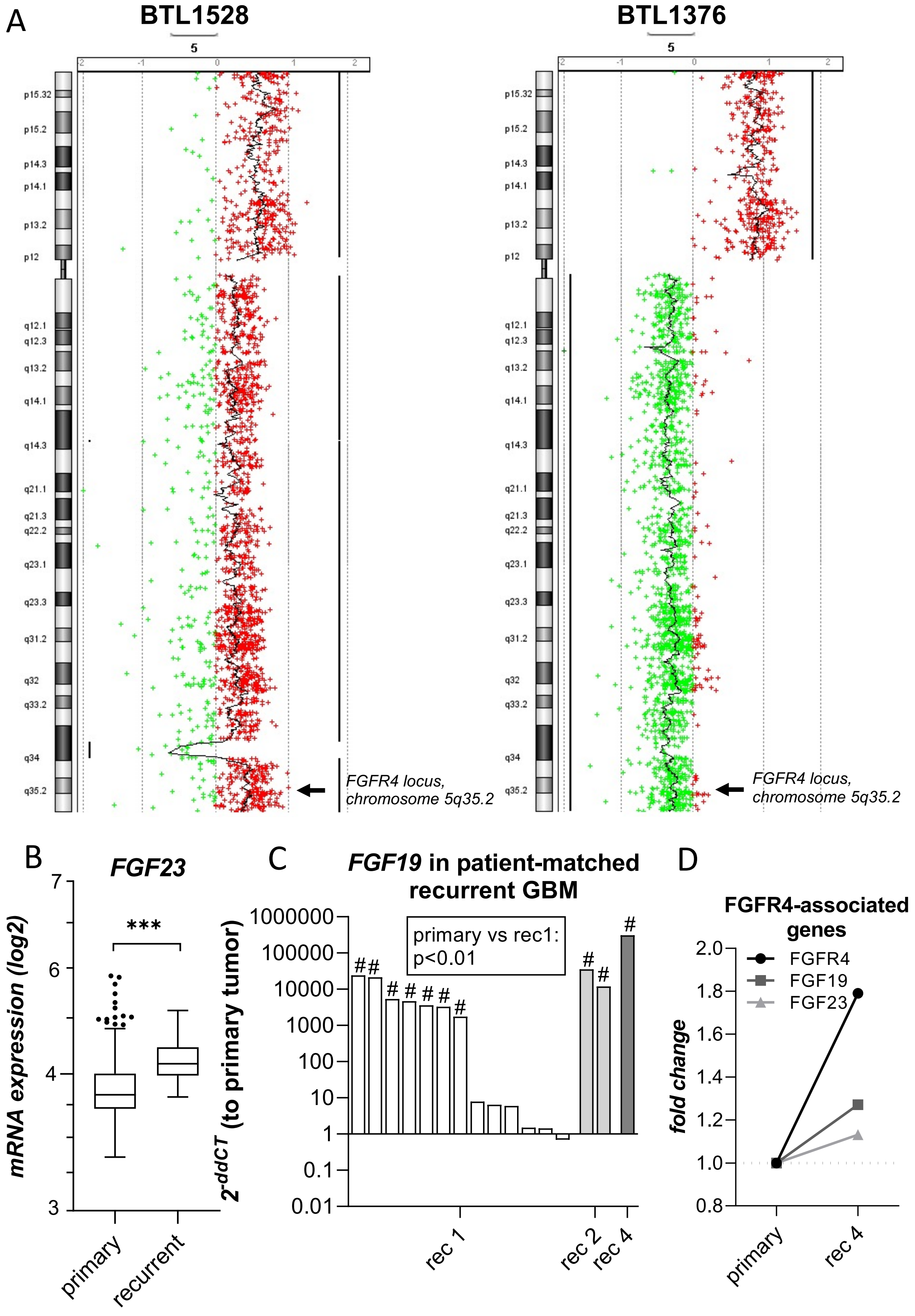

A
BTL1528
BTL1376
FGFR4 locus, chromosome 5q35.2
FGFR4 locus, chromosome 5q35.2
B
FGF23
mRNA expression (log2)
***
primary
recurrent
C
FGF19 in patient-matched recurrent GBM
primary vs rec1: p<0.01
$2^{-ddCT}$ (to primary tumor)
rec 1
rec 2
rec 4
D
FGFR4-associated genes
FGFR4
FGF19
FGF23
fold change
primary
rec 4

**Supplementary Figure S3.** ***FGFR4**$^{high}$* **GBM models lack *FGFR4* gene dose alterations, but *FGFR4* ligand genes are overexpressed in recurrent GBM. (A)** Chromosome 5 profiles from array comparative genomic hybridization of *FGFR4*$^{high}$ GBM cells BTL1528 and BTL1376 are shown. The gene dose (log2 ratio of tumor DNA/non-malignant DNA) is depicted. The arrow indicates the chromosomal locus of *FGFR4*. red = gains, green = losses. **(B)** *FGF23* mRNA expression in primary (n=497) and recurrent GBM (n=16) was analyzed in the TCGA-GBM-HG-U133A data set. Statistical significance was analyzed by Student's t-test. *** p<0.001 **(C)** *FGF19* mRNA levels were determined by qRT-PCR patient-matched primary GBM (n=14) and sequential recurrences (rec1, n=13; rec2, n=2; rec4, n=1). Statistical analyses: Student's t-test. # indicate recurrent lesions matching to primary tumors with undetectable *FGF19* expression after 50 cycles (CT >50), thus, arbitrarily set to 50 for normalization approaches. **(D)** mRNA expression levels of *FGFR4* and associated ligand genes *FGF19* and *FGF23* derived from whole genome gene expression arrays are shown in primary tumor (set to 1) and the 4$^{th}$ recurrence of one GBM patient.

# Figure S4

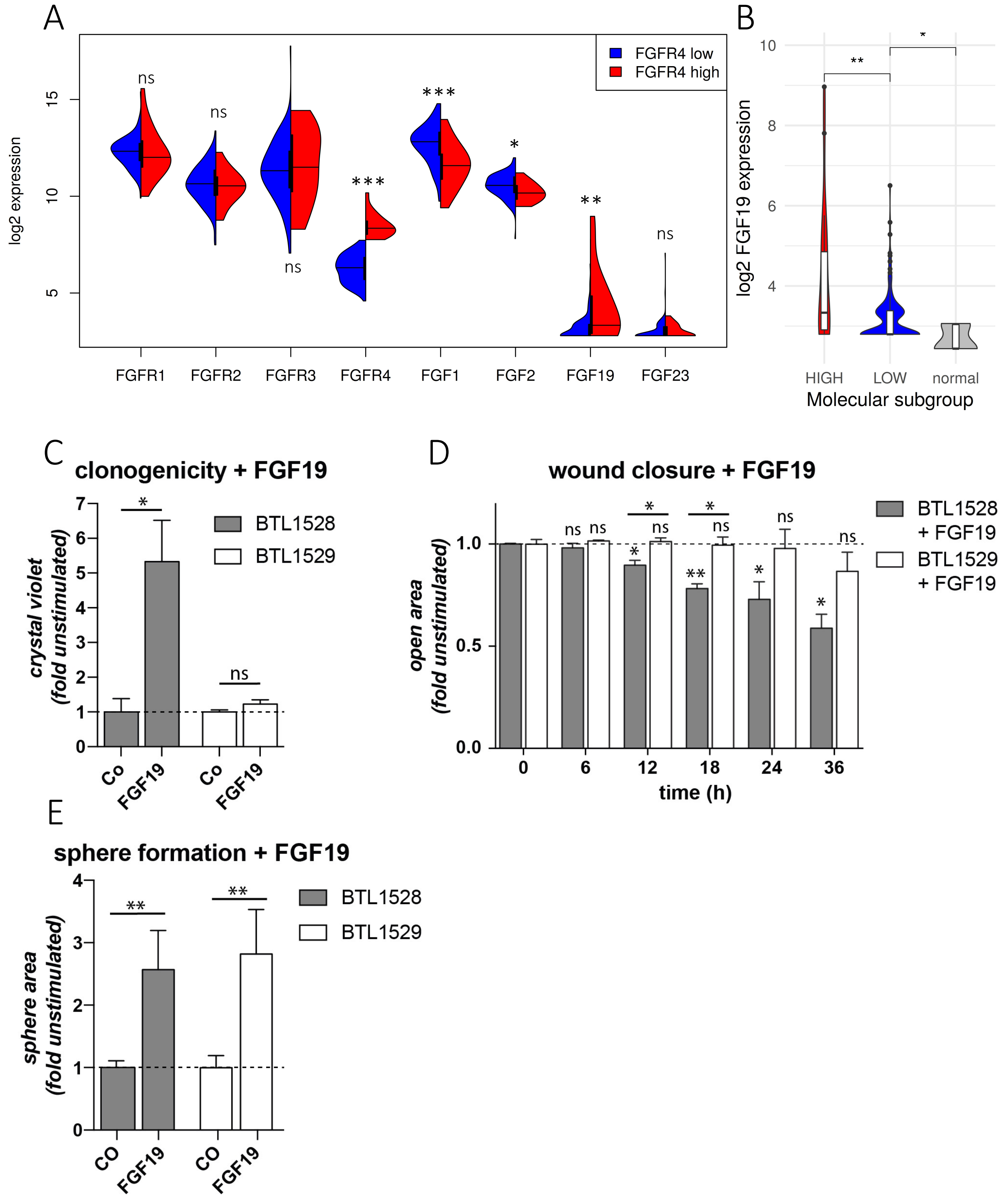

A
FGFR4 low
FGFR4 high
log2 expression
ns
ns
ns
***
***
*
**
ns
FGFR1
FGFR2
FGFR3
FGFR4
FGF1
FGF2
FGF19
FGF23
B
log2 FGF19 expression
**
*
HIGH
LOW
normal
Molecular subgroup
C
clonogenicity + FGF19
crystal violet (fold unstimulated)
BTL1528
BTL1529
Co
FGF19
D
wound closure + FGF19
open area (fold unstimulated)
BTL1528 + FGF19
BTL1529 + FGF19
time (h)
E
sphere formation + FGF19
sphere area (fold unstimulated)
BTL1528
BTL1529
CO
FGF19

**Supplementary Figure S4. FGF19 promotes hallmarks of GBM aggressiveness. (A+B)** TCGA-GBM RNA sequencing data were analyzed and stratified into *FGFR4*$^{high}$ (red) and *FGFR4*$^{low}$ (blue) subgroups. **(A)** mRNA expression levels of the indicated FGF/FGFR family members in the *FGFR4*$^{high}$ (red) and *FGFR4*$^{low}$ (blue) GBM subgroups are depicted as violin plots (median ± SD). **(B)** *FGF19* expression levels are depicted in the indicated GBM subgroups and in non-malignant brain (grey). **(C)** Clonogenicity, **(D)** wound-healing assays, **(E)** and sphere formation assays upon stimulation with FGF19 are shown. Endogenously *FGFR4*$^{high}$ BTL1528 and *FGFR4*$^{low}$ BTL1529 GBM cells were seeded in serum-supplemented growth medium. After 24h, serum was deprived in **(C)** and cells were stimulated with FGF19-conditioned medium (50ng/ml) in **(C-E,** mean +/- SEM**)**. Effects were observed after 7 days **(C)**, over time as indicated **(D)**, or after 4 days **(E)**. Statistics: **(A+B)** Wilcoxon signed-rank test; **(C)** Student's t-tests; **(D+E)** 2-way ANOVA; * $p<0.05$, ** $p<0.01$, *** $p<0.001$, ns = not significant;

# Figure S5

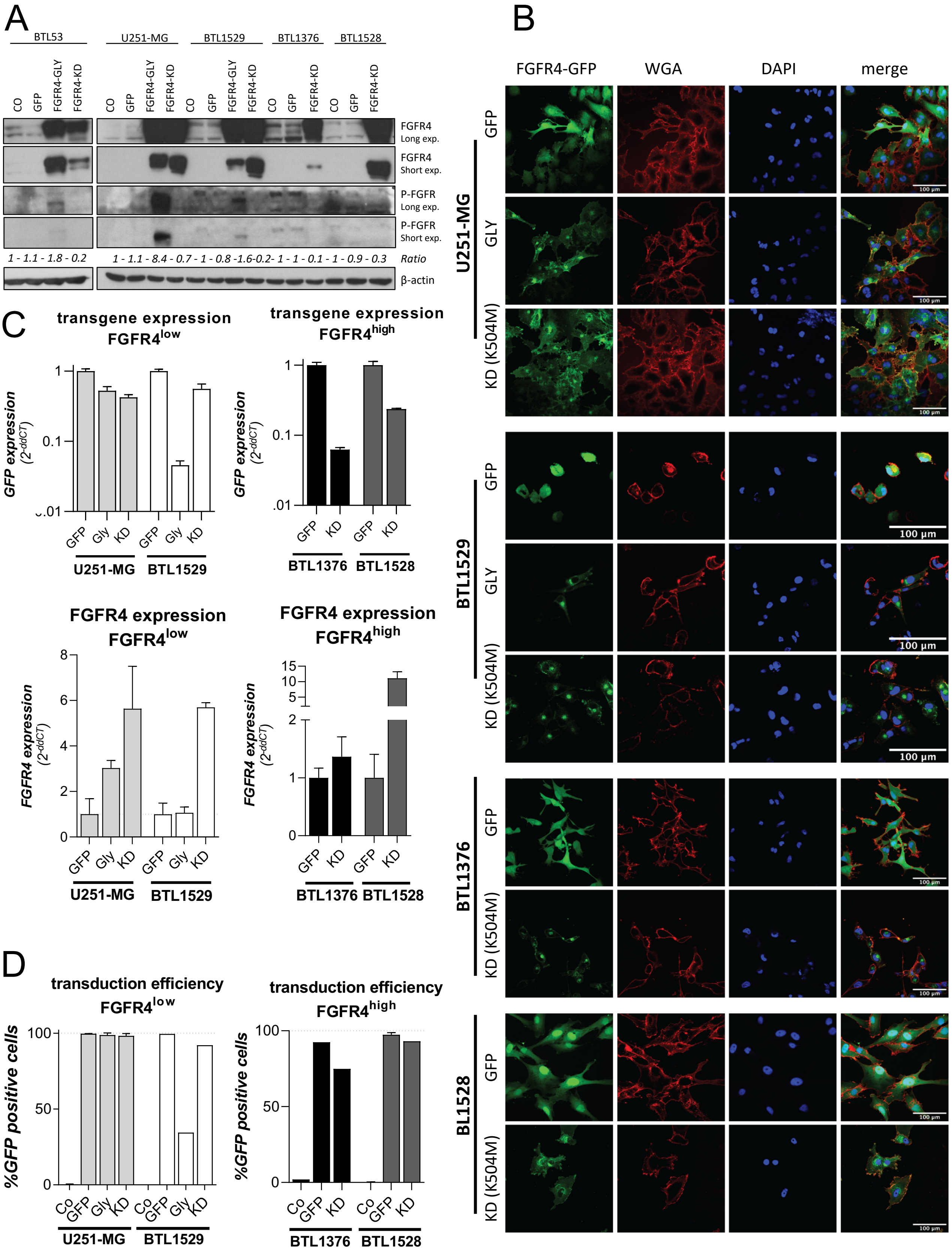

A
BTL53
U251-MG
BTL1529
BTL1376
BTL1528
CO
GFP
FGFR4-GLY
FGFR4-KD
FGFR4 Long exp.
FGFR4 Short exp.
P-FGFR Long exp.
P-FGFR Short exp.
1 - 1.1 - 1.8 - 0.2
1 - 1.1 - 8.4 - 0.7 - 1 - 0.8 -1.6-0.2- 1 - 1 - 0.1 - 1 - 0.9 - 0.3
Ratio
β-actin
B
FGFR4-GFP
WGA
DAPI
merge
U251-MG
BTL1529
BTL1376
BL1528
GFP
GLY
KD (K504M)
100 µm
C
transgene expression FGFR4low
transgene expression FGFR4high
GFP expression (2-ddCT)
FGFR4 expression FGFR4low
FGFR4 expression FGFR4high
FGFR4 expression (2-ddCT)
GFP
Gly
KD
D
transduction efficiency FGFR4low
transduction efficiency FGFR4high
%GFP positive cells
Co

**Supplementary Figure S5. Wild-type and kinase-dead FGFR4 transduction results in stably overexpressing GBM cell models.** BTL1528 and BTL1376 (*FGFR4$^{high}$*) as well as BTL53, U251-MG, and BTL1529 (*FGFR4$^{low}$*) cells were transduced with retroviral constructs resulting in stable sublines overexpressing *GFP, FGFR4-Gly-GFP* or *FGFR4-KD(K504M)-GFP*. **(A)** FGFR4 expression and phosphorylation of FGFR proteins of the respective FGFR4-variant subclones and GFP-only-transduced cells (GFP) as compared to untransduced controls (Co) were analyzed by Western blotting of total protein lysates. β-actin served as loading control. Blots were normalized to the respective β-actin signal, and subsequently the ratios were calculated to show pathway activity in every cell model. **(B)** Confocal photomicrographs of the modified *FGFR4-GFP* or *GFP*-only cells as indicated counterstained with wheat germ agglutinin (WGA) and DAPI for plasma membrane and nuclear stain, respectively, are shown. Scale bars indicate 100µm. **(C)** *GFP (upper panel)* and *FGFR4 (lower panel)* mRNA expression levels are shown indicating the modified *FGFR4-GFP* transgenes in endogenously *FGFR4$^{low}$ (left panel)* and *FGFR4$^{high}$* cells *(right panel).* Data are given as mean +/- SEM from three independent experiments normalized to *GFP*-only cells, set to 1 ($2^{-ddCT}$). **(D)** Transduction efficiency upon *FGFR4-Gly-GFP*, *FGFR4-KD (K504M)-GFP* or *GFP*-only overexpression was analyzed by flow cytometry testing for GFP-positive cells in endogenously *FGFR4$^{low}$ (left panel)* and *FGFR4$^{high}$ (right panel)* GBM models. Data are given as mean +/- SD from two independent experiments. GLY= *FGFR4-388Gly-GFP*, KD = *FGFR4 kinase-dead (K504M)-GFP*

# Figure S6

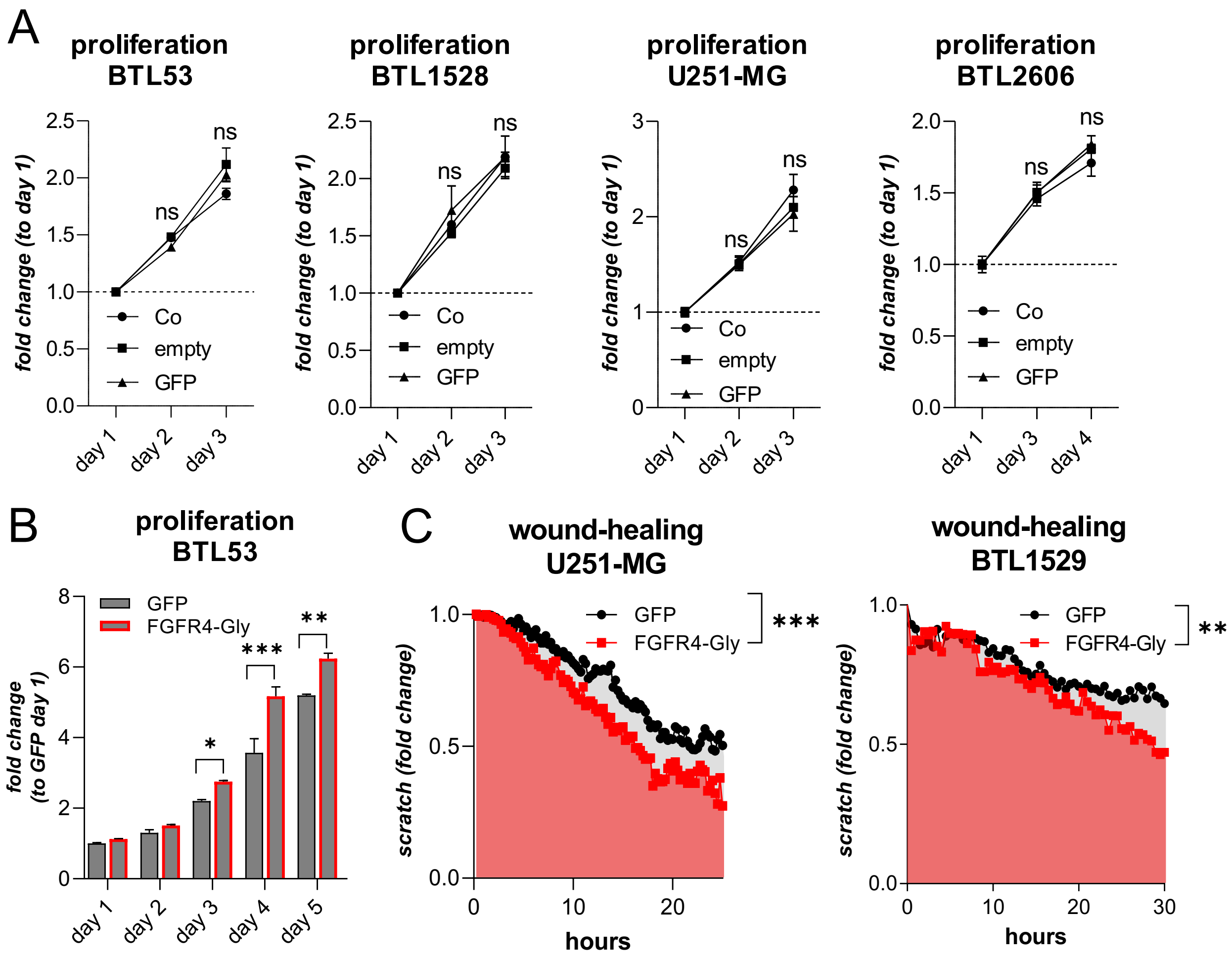


**Supplementary Figure S6. *FGFR4* overexpression promotes proliferation and wound healing capacity of endogenously *FGFR4*$^{low}$ GBM cells. (A)** Proliferation capacity of control cells, as compared to stable empty-vector- and GFP-transduced GBM subclones. ns=not significant. **(B)** Proliferation capacity of *FGFR4-Gly*-overexpressing BTL53 GBM cells versus *GFP*-transduced sublines is shown. Two-way ANOVA: *p<0.05, **p<0.01, ***p<0.001 **(C)** Wound-healing capacities of *FGFR4-Gly*- and *GFP*-transduced control sublines of U251-MG *(left panel)* and BTL1529 *(right panel)* are depicted. One representative experiment per cell line is shown. Two-way ANOVA: **p<0.01,*** p<0.001

# Figure S7

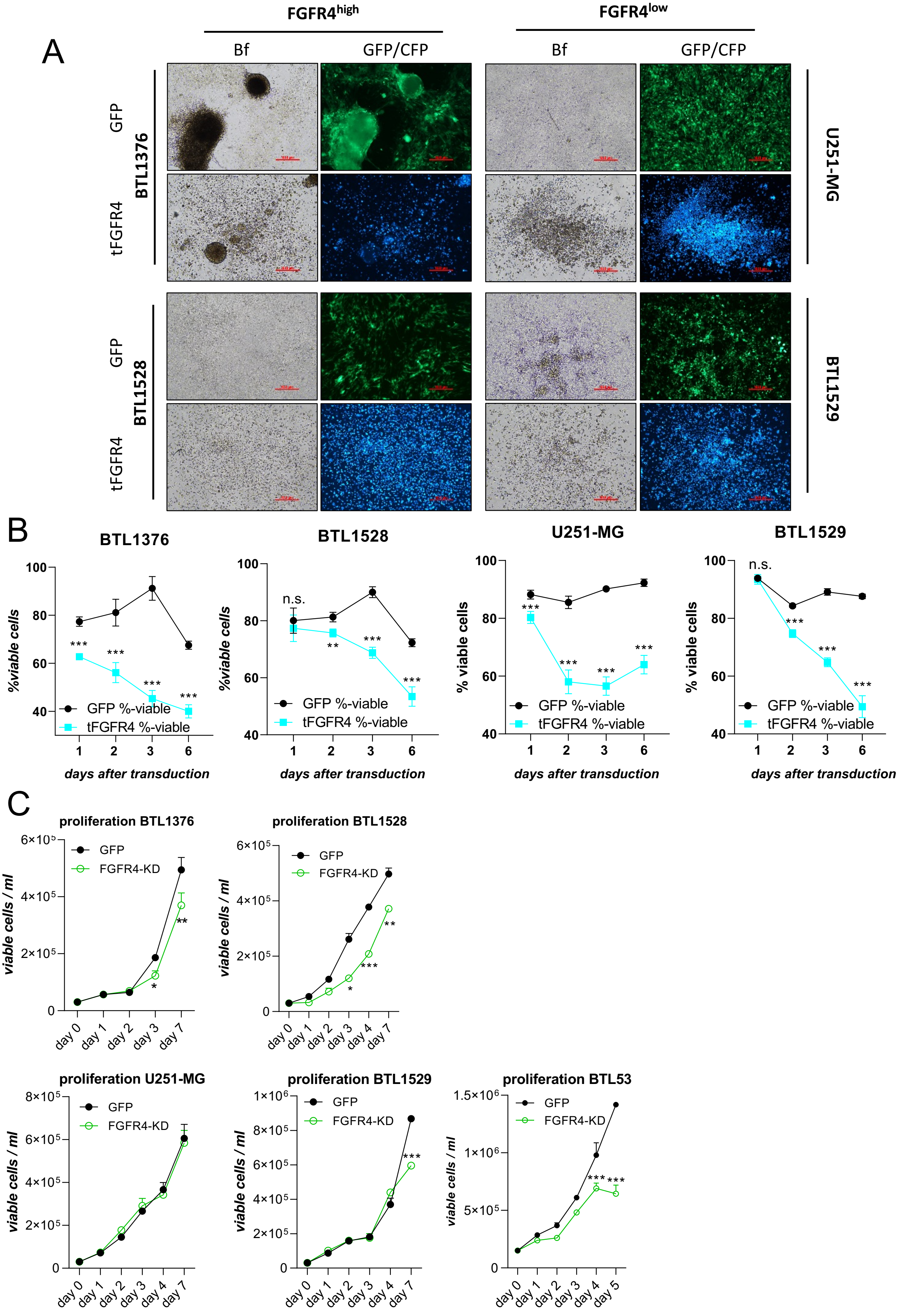

A
FGFR4high
FGFR4low
Bf
GFP/CFP
Bf
GFP/CFP
GFP
tFGFR4
BTL1376
GFP
tFGFR4
BTL1528
U251-MG
BTL1529
B
BTL1376
BTL1528
U251-MG
BTL1529
%viable cells
% viable cells
days after transduction
GFP %-viable
tFGFR4 %-viable
n.s.
C
proliferation BTL1376
proliferation BTL1528
proliferation U251-MG
proliferation BTL1529
proliferation BTL53
viable cells / ml
GFP
FGFR4-KD
day 0
day 1
day 2
day 3
day 4
day 5
day 7

**Supplementary Figure S7. Anti-proliferative effects of FGFR4 inactivation in GBM cells.** Viability/proliferation assays of the indicated cell models upon *tFGFR4* **(A+B)**, *FGFR4-KD (K504M)* **(C)** or *GFP* transduction were performed for the indicated time frame. **(A)** Representative photomicrographs after 6 days of *tFGFR4* (CFP) or *GFP* transduction are depicted. Scale bars indicate 1000µm. Bf = bright field. **(B+C)** The percentage of viable cells, measured by CASY cell counter, are depicted at the indicated time points. Results are given as mean +/- SD from one representative out of three independent experiments performed in duplicates. **(C)** Significance levels in **(B+C)** were quantified using 2-way ANOVA with Bonferroni correction. * $p<0.05$, ** $p<0.01$, *** $p<0.001$, n.s. = not significant

# Figure S8

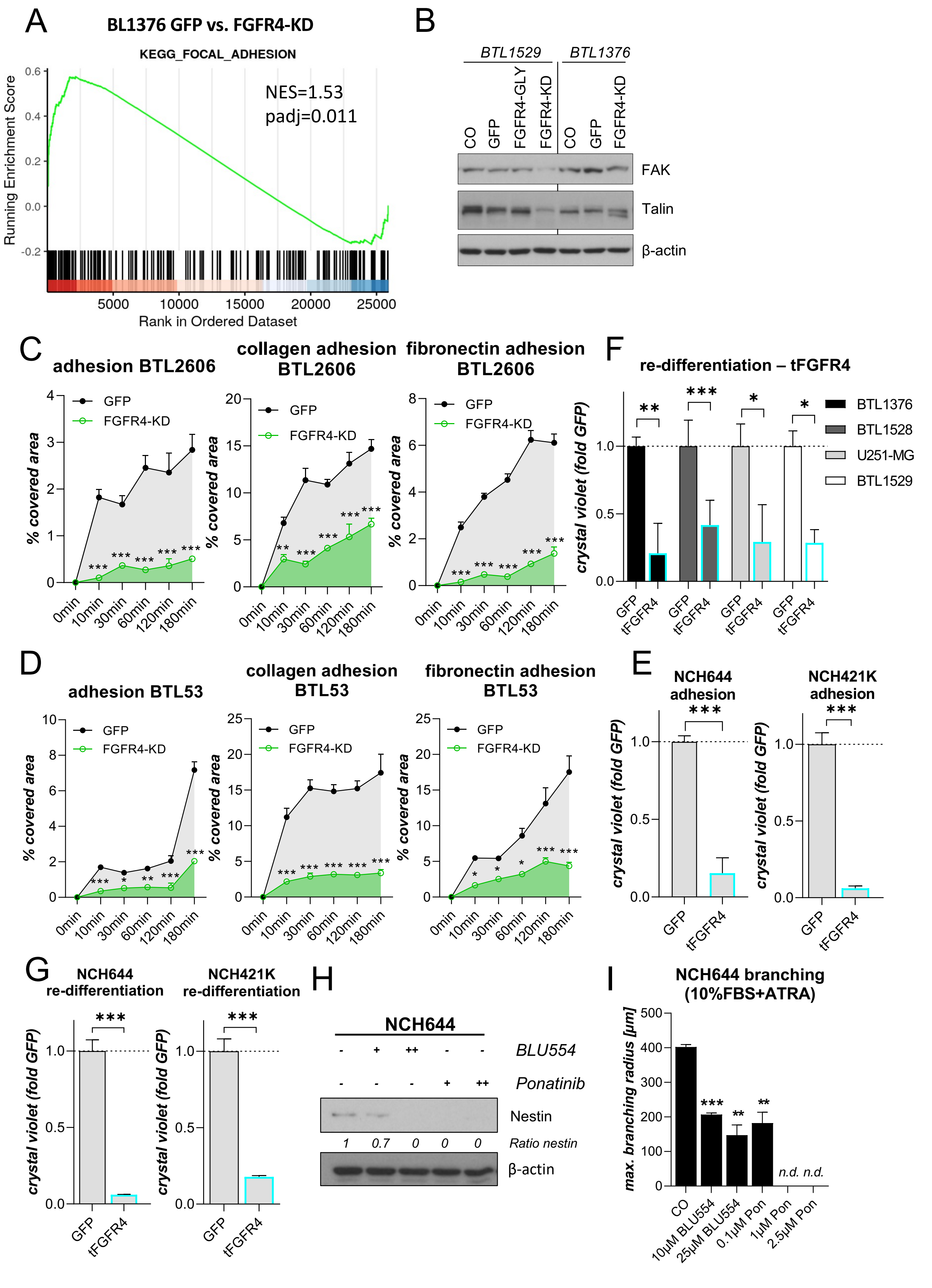
A
BL1376 GFP vs. FGFR4-KD
KEGG_FOCAL_ADHESION
NES=1.53
padj=0.011
Running Enrichment Score
Rank in Ordered Dataset
B
BTL1529
BTL1376
CO
GFP
FGFR4-GLY
FGFR4-KD
FAK
Talin
β-actin
C
adhesion BTL2606
collagen adhesion BTL2606
fibronectin adhesion BTL2606
GFP
FGFR4-KD
% covered area
0min
10min
30min
60min
120min
180min
D
adhesion BTL53
collagen adhesion BTL53
fibronectin adhesion BTL53
F
re-differentiation – tFGFR4
BTL1376
BTL1528
U251-MG
BTL1529
crystal violet (fold GFP)
tFGFR4
E
NCH644 adhesion
NCH421K adhesion
G
NCH644 re-differentiation
NCH421K re-differentiation
H
NCH644
BLU554
Ponatinib
Nestin
Ratio nestin
1
0.7
0
0
0
β-actin
I
NCH644 branching (10%FBS+ATRA)
max. branching radius [µm]
CO
10µM BLU554
25µM BLU554
0.1µM Pon
1µM Pon
2.5µM Pon
n.d.
n.d.

**Supplementary Figure S8. FGFR4 blockade impairs focal adhesion capacity of glioma cells and GSCs. (A)** GSEA revealed altered expression profiles of genes associated to the *KEGG Focal Adhesion* pathway of BTL1376 *GFP* versus *FGFR4-KD (K504M)* sublines. NES = normalized enrichment score, padj = adjusted p-value. **(B)** FAK and talin protein expression levels of *FGFR4-Gly-, FGFR4-KD(K504M)- and GFP*-transduced GBM models, as well as untransduced controls (Co) were detected by Western blot analyses. β-actin served as loading control. **(C-E)** Adhesion capacity towards cell culture polystyrene of the endogenously *FGFR4*$^{low}$ BTL2606 and BTL53 GBM models **(C+D** left panels**)** and NCH644 and NCH421K GSC models **(E)** was analyzed upon *tFGFR4* or *GFP* transduction. Additionally, adhesion capacity of GBM models towards collagen **(C+D** middle panels**)** or fibronectin **(C+D** right panels**)** was analyzed. **(F+G)** Re-differentiation capacities of the indicated GBM **(F)** and GSC **(G)** spheroids upon FGFR4 inactivation using the *tFGFR4* adenovirus were evaluated and compared to *GFP*-transduced controls (set to 1). Cells were transduced with the respective adenovirus (100moi) and kept under serum-deprived conditions. After 5 days, spheres were re-plated in serum-supplemented growth medium. Re-differentiation capacity of the GBM spheroids after another 5 days was analyzed using crystal violet staining. Results are given as mean +/- SEM from three independent experiments. **(H)** Nestin expression was analyzed in NCH644 cells treated with 10µM (+) or 25µM (++) BLU554, or 1µM (+) / 2.5µM (++) ponatinib for 20h by Western blots. β-actin served as loading control. Ratios indicate expression normalized to β-actin. **(I)** Differentiation capacity of the GSC model NCH644 was analyzed upon stimulation with serum-supplemented medium and all-trans retinoic acid (ATRA). Cells were seeded as single cells in GSC medium and treated with the FGFR4-targeting inhibitors BLU554 or ponatinib (Pon) as indicated. After 72h, spheroids were re-seeded in serum-supplemented medium with ATRA and re-treated as indicated. Microphotographs were taken after 72h and results are given as maximal radius from sphere center (µm, mean +/- SD) from two independent experiments performed in duplicates. Significance levels were quantified using 2-way ANOVA with Bonferroni correction **(C,D,F,I)** or Student's t-tests **(E,G)**: * p<0.05, ** p<0.01, *** p<0.001, n.d. not detected.

# Figure S9

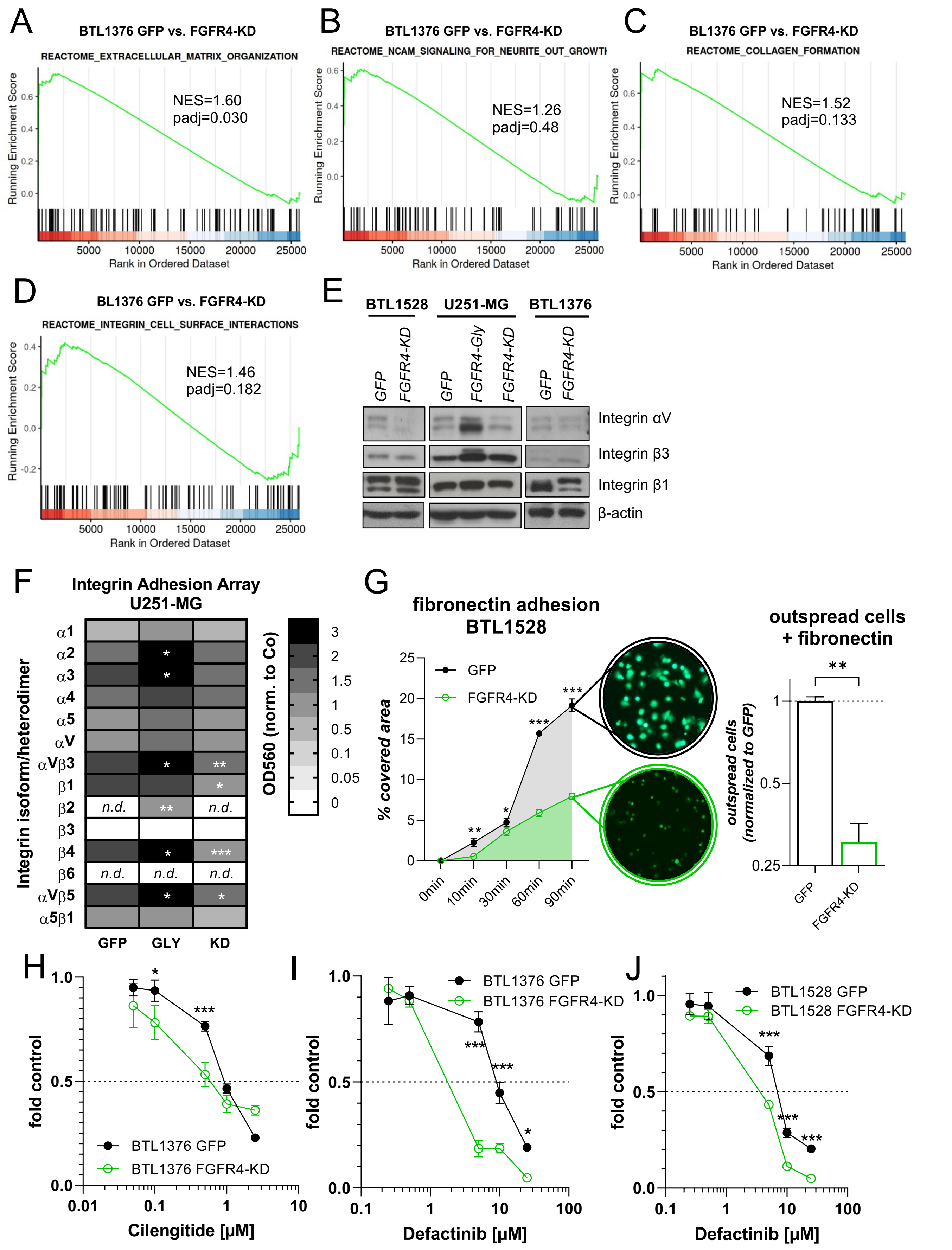

A
BTL1376 GFP vs. FGFR4-KD
REACTOME_EXTRACELLULAR_MATRIX_ORGANIZATION
NES=1.60
padj=0.030
Running Enrichment Score
Rank in Ordered Dataset
B
BTL1376 GFP vs. FGFR4-KD
REACTOME_NCAM_SIGNALING_FOR_NEURITE_OUT_GROWTH
NES=1.26
padj=0.48
C
BL1376 GFP vs. FGFR4-KD
REACTOME_COLLAGEN_FORMATION
NES=1.52
padj=0.133
D
BL1376 GFP vs. FGFR4-KD
REACTOME_INTEGRIN_CELL_SURFACE_INTERACTIONS
NES=1.46
padj=0.182
E
BTL1528
U251-MG
BTL1376
GFP
FGFR4-KD
FGFR4-Gly
Integrin αV
Integrin β3
Integrin β1
β-actin
F
Integrin Adhesion Array
U251-MG
Integrin isoform/heterodimer
OD560 (norm. to Co)
n.d.
GFP
GLY
KD
G
fibronectin adhesion
BTL1528
GFP
FGFR4-KD
% covered area
0min
10min
30min
60min
90min
outspread cells
+ fibronectin
outspread cells
(normalized to GFP)
H
fold control
BTL1376 GFP
BTL1376 FGFR4-KD
Cilengitide [µM]
I
BTL1376 GFP
BTL1376 FGFR4-KD
Defactinib [µM]
J
BTL1528 GFP
BTL1528 FGFR4-KD
Defactinib [µM]

**Supplementary Figure S9. FGFR4 inactivation drives loss of integrin and collagen networks in GBM resulting in decreased fibronectin adhesion. (A-D)** GSEA of BTL1376 *GFP* versus *FGFR4-KD (K504M)* gene expression data revealed altered expression of genes involved in the depicted *REACTOME* pathways. NES = normalized enrichment score, padj = adjusted p-value. **(E)** Integrin αV, β3 and β1 protein expression levels of the indicated cell models and sublines were analyzed by Western blotting. β-actin served as loading control. **(F)** Integrin-mediated cell adhesion arrays of the endogenously *FGFR4*$^{low}$ GBM model U251-MG as compared to the respective FGFR4-GLY- (GLY) and FGFR4-KD-transduced (KD) subclones are shown. Cell adhesion was analyzed by absorbance measurement following cell staining in duplicates. Statistical significance was tested by 2-way ANOVA with Bonferroni correction. **(G)** Adhesion capacity towards fibronectin was analyzed in BTL1528 *FGFR4-KD (K504M)*- and *GFP*-transduced cells. Plates were coated with fibronectin and cells were incubated for the indicated time points. The percentage of the area coated by cells is given as mean +/- SD from one representative out of three independent experiments *(left panel)*. Significance levels were quantified using 2-way ANOVA with Bonferroni correction. Photomicrographs of time point 90min are shown exemplarily. The number of outspread, fully attached cells was counted and data are given relative to the *GFP*-transduced controls for time point 90min *(right panel).* Student's t-test was performed. **(H-J)** Impact of treatment with cilengitide **(H)** or defactinib **(I+J)** as indicated on the viability of BTL1376 **(H+I)** and BTL1528 **(J)** *FGFR4-KD(K504M)*- and *GFP*-transduced GBM sublines is shown. One representative out of three independent experiments in triplicates is depicted (mean of triplicates ± SD). 2-way ANOVA with Bonferroni correction was performed. In all cases: n.d. = not detected, * $p<0.05$, ** $p<0.01$, *** $p<0.001$.

# Figure S10

A

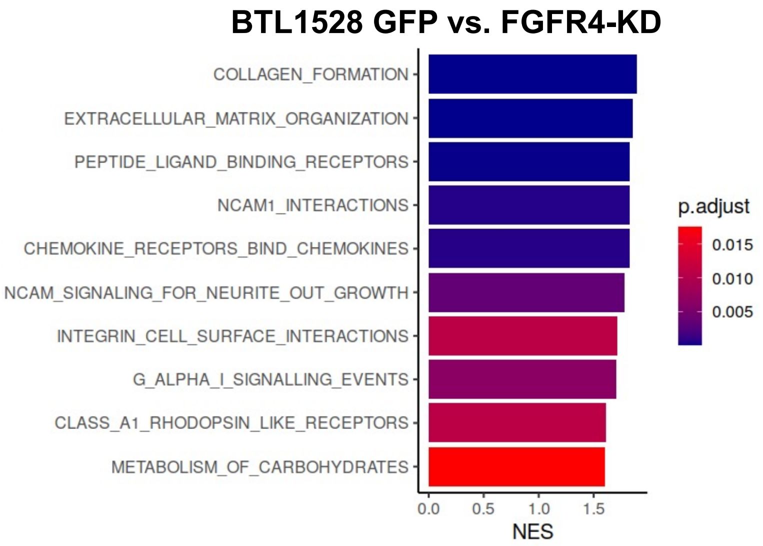


B

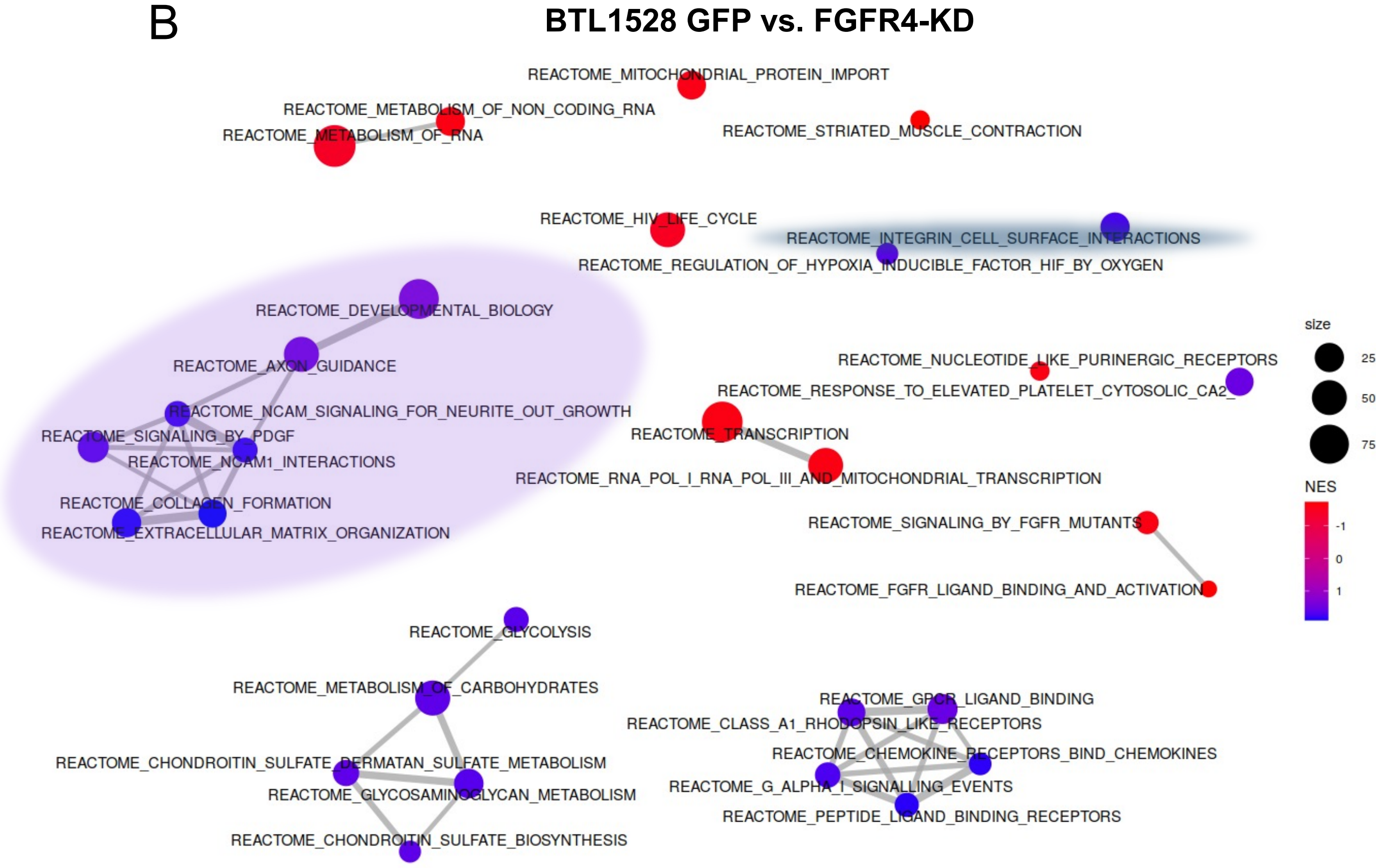


**Supplementary Figure S10. Gene ontologies related to FGFR4 blockade cluster in functional modules. (A)** The top 10 (ranked by NES) *REACTOME* ontologies in BTL1528 *GFP*- versus *FGFR4-KD(K504M)*-transduced subclones are depicted as bar plot. **(B)** Enrichment map of the top 30 (ranked by NES, p-adjust<0.05) *REACTOME* gene ontologies in BTL1528 *GFP*- versus *FGFR4-KD(K504M)*-expressing GBM sublines is illustrated. Edge size indicates the grade of overlap in the respective gene sets. Node size indicates number of altered genes per gene set. NES=normalized enrichment score.
