# Supplementary material for: Fibroblast growth factor receptor 4 promotes glioblastoma progression: a central role of integrin-mediated cell invasiveness
Source: Acta Neuropathol Commun. 2022 Apr 28;10:65. doi: 10.1186/s40478-022-01363-2 (PMC9052585; doi:10.1186/s40478-022-01363-2)
Supplement: Supplementary file 2 — Additional file2. Supplementary Materials [file 40478_2022_1363_MOESM2_ESM.docx]

**Supplementary materials**

**Cell cultures conditions**

Primo-GBM cells were cultured in RPMI-1640 medium (Sigma-Aldrich) supplemented with 10% fetal calf serum (FCS, Gibco Thermo Fisher Scientific, Waltham, Massachusetts, USA). GSC models were cultured in DMEM/F-12 medium supplemented with 20% BIT-100 serum-free supplement (ProVitro, Berlin, Germany), EGF (Sigma-Aldrich), and bFGF (PeptroTech, Rocky Hill, NJ, USA) (each 20ng/mL). All cells were cultured under humidified conditions at 37°C/5% CO_2_ and regularly checked for *Mycoplasma* contamination. No anti-microbial substances were used. All cells were re-established from frozen stocks every six weeks to avoid changes during cell culture propagation.

**FGFR4 organ distribution data in non-malignant tissues**

*FGFR4* expression in different organs was analyzed using the RNA sequencing GTEx data set. The Genotype-Tissue Expression (GTEx) Project was supported by the Common Fund of the Office of the Director of the National Institutes of Health, and by NCI, NHGRI, NHLBI, NIDA, NIMH, and NINDS. The data used for the analyses described in this manuscript were obtained from the GTEx Portal on 08/10/20, dbGaP accession number is phs000424.v8.p2. *FGFR4* mRNA expression levels (mRNA expression array probe A_23_P92754) were compared in different adult non-malignant brain areas. Data for this purpose were obtained from the Allen brain atlas (brain-map.org).

**Survival data**

Cutpoint determination for sample stratification based on the *FGFR4* expression values was performed by maximizing the log-rank statistics (*surv_cutpoint()* function from *survminer* package in R). This yielded an *FGFR4*-high subset of 13% (50/380) from the whole cohort. Kaplan Meier survival analysis was applied using this patient stratification for the mas5.0 normalized TCGA-GBM-HG-U133A dataset (Figure S2). Accordingly, a “hard” cutoff was applied to the REMBRANDT dataset, again stratifying the highest-expressing 13% of GBM as *FGFR4*-high (Figure 1B). Log-rank tests were used for significance calculations.

**The REMBRANDT data collection**

The REMBRANDT mRNA expression set was downloaded (GSE108474, download on 11/05/2020, n=541) and data were processed using R environment (v.4.0.0). Data were normalized by applying the *rma()* function of the *affy* package. *FGFR4* levels were plotted according to the DISEASE_TYPE variable in the data set for Figure 1A (non-tumor: n=28; GBM: n=219). In order to focus on therapy-naïve GBM patients, the variable PRIOR_THERAPY_SURGERY_TUMOR_HISTOLOGY was used to analyze the relevance of FGFR4 in GBM patient survival (n=99).

**Data collection, processing, normalization of RNA sequencing data**

The RNA sequencing *in silico* data used in this study was obtained from the TCGA-GBM project (https://portal.gdc.cancer.gov/projects/TCGA-GBM), and data analysis was performed using R statistical environment (v.4.0.0). HTSeq raw counts were downloaded using the GDC API and yielded a total of 174 patient samples, comprising 169 tumor and 5 non-tumor tissue samples. In a preprocessing step, genes with counts of less than 10 were removed. Further analysis was carried out using the well-established DESeq2 pipeline (package *DESeq2,* ^1^). Counts were normalized by applying the *estimateSizeFactors()* function to all samples. This normalization approach corrects for differences in library size as well as library composition and therefore facilitates comparison of independent samples. Following normalization, non-tumor tissue samples were extracted from the combined data set and henceforth treated individually.

**Stratification, differentially expressed genes (DEG), gene set enrichment analysis (GSEA)**

The stratification obtained from maximization of the rank statistics as described above was further applied to the RNA sequencing data, resulting in n=22 (13%) in the *FGFR4^high^* and n=147 (87%) patients in the *FGFR4^low^* subgroups*.* DEG between both groups (*FGFR4^high^ versus* *FGFR4^low^*) were determined using DESeq2. Only genes displaying a |logFC| > 1 and FDR < 0.05 (Benjamini-Hochberg) were considered as differentially expressed. GSEA of gene ontology terms was performed using the *fgsea* package ^2^.
A list of genes ranked according to their log fold change (FC) served as input for the function *gseGO(),* which used the *org.Hs.eg.db* annotation package as a reference to identify significantly enriched biological processes (BP). To further expand the biological scope, a second GSEA was performed referencing to the molecular signatures C2 database (v7.1). Default GSEA parameters were used with exception of exponent=1.5 and minGSSize=15. For dot plots in Figure 2B, Gene Ratios were calculated as leadingEdge / size of the respective gene set.

## mRNA expression microarray and array comparative genomic hybridization (aCGH)

RNA was isolated using the RNeasy Mini Kit (QIAGEN, Hilden, Germany) following the manufacturer’s protocol. Whole genome mRNA expression microarrays (Agilent) were performed as described in ^3^ and according to the protocols provided by the manufacturer. DEG were extracted using the LIMMA package of R ^4^. GSEAs were performed as described above. Isolation of genomic DNA and aCGH analysis was performed as described ^5^. Labeling and hybridization procedures were performed according to the manufacturer’s instructions using the SureTag DNA Labeling Kit (Agilent Technologies). Microarrays were scanned using the G2505B Micro Array Scanner (Agilent Technologies). Following feature extraction (Feature Extraction software form Agilent) data analysis for aCGH and mRNA expression was carried out using Agilent Genomic Workbench or GeneSpring softwares, respectively (both Agilent Technologies).

**Immunohistochemistry (IHC) and histology**

Formalin fixed and paraffin embedded (FFPE) patient-derived tumor samples were cut in 2µm slices and stained using hematoxylin and eosin (HE) (Sigma-Aldrich). Consecutive slides of every sample were stained with an FGFR4–targeting antibody (Cell Signaling Technology, Baverly, Massachusetts, USA, #D3B12 XP – 1:500). Whole FFPE mouse brains were transversally cut in cycles of five consecutive 2.5µm slices every 75µm. Consecutive sections of every level were stained using HE and a GFP-targeting antibody (Cell Signaling Technology, #2555 – 1:250 in case of BTL1528 FGFR4-KD-GFP-impanted brains or 1:1000 in case of BTL1528 GFP-implanted brains).

Precisely, slides were deparaffinized, hydrated, and subsequently treated with 0.3% H_2_O_2_ in TBS for 10min. Antigen retrieval was achieved by wet autoclave treatment. Samples were washed in TBS-Tween (0.1%) and antibody development was performed using the Lab Vision™ UltraVision™ LP Detection System: HRP Polymer/DAB Plus Chromogen (Thermo Fisher Scientific), according to manufacturer’s protocols. FGFR4 antibody was diluted 1:500 and GFP antibody 1:250-1:1000 in antibody diluent (Cell Signaling Technology, #8112) supplemented with goat serum (1:100, Dako - Agilent, #X0907) for 1h at room temperature. Slides were counterstained with Hematoxylin Gill III (Merck, #1.05174.0500) and, following dehydration, covered in Entellan (Merck, #1.07961.0100). Furthermore, tissues of tumor xenografts from U251-MG and BTL1528 were shock frozen on dry ice, and 5µm cryo-sections were produced. Subsequently, samples were fixed in 4% paraformaldehyde for 10min at room temperature and covered with Vectashield with 4′,6-diamidino-2-phenylindole (DAPI, Vector laboratories, Burlingame, California, USA).

**Protein isolation and Western blot**

Membrane-enriched fractions were isolated as described previously ^6^. For protein isolation of subcutaneous tumors, fresh explants were shock frozen in liquid nitrogen after termination of the mice. Tumor tissue was thawed on ice and mechanically crushed in protein lysis buffer^7^, followed by ultrasound. Protein isolation and analyses by Western blot were performed as previously described ^7^. In short, 15µg of proteins were loaded onto sodium dodecyl sulfate - polyacrylamide gels and electrophoresis was run. All antibodies were diluted in 3% bovine serum albumin in TBS-Tween (0.1%). Hep3B, a hepatocellular carcinoma (HCC) model, exhibiting high FGFR4 levels and harboring an amplification of the FGFR4 ligand gene *FGF19* ^8^, was used as positive control in screening blots. Protein isolation and subsequent Western blots of all *in vitro* experimental settings were performed at least three times. Table S1 lists all used antibodies and the respective further dilution information. β-actin served as loading control. Blots were densitometrically quantified by Fiji software. Ratios were calculated as outlined in the corresponding figure legends.

Generation of retroviral constructs

The full-length *FGFR4-388Gly* and the *FGFR4-KD (K504M)* gene variants were PCR-amplified using the following Infusion (IFU) primers (1µM):

Forw. FGFR4 IFU: 5’-GGCCTCGTACGCTTACCATGCGGCTGCTGCTG-3’

Rev. FGFR4 IFU: 5’-ATGGTGGCGATGGATTCTGTCTGCACCCCAGACC-3’

Q5 high-fidelity DNA Taq polymerase kit (#E0555L, New England Biolabs, Ipswich, Massachusetts, USA) was used for PCR-amplification. PCR resulted in generation of a full-length *FGFR4* product with 15bp extensions complementary to the restriction sites of the linearized target vector, as suggested in the In-Fusion HD cloning kit (Takara Bio Inc., ‎Shimogyō-ku, Kyoto, Japan). The amplification product was isolated from a 0.8% agarose gel and cleaned up using Monarch DNA gel extraction kit (New England Biolabs, Ipswich, Massachusetts, USA) according to the manufacturer’s instructions. The target vector pQCXIP-EGFP contains one single BamHI restriction site close to the multiple cloning site, upstream of the *EGFP* gene. Furthermore, the vector contains an ampicillin resistance and a puromycin mammalian selection cassette, both downstream of the *EGFP* gene, which is driven by an CMV promoter upstream the BamHI restriction site. This vector was furthermore used as control vector for the generation of GFP-only cell models. The In-Fusion HD Cloning (Takara Bio Inc.) was performed according to developer’s protocols to specifically clone the *FGFR4* gene variants into the linearized (BamHI restriction, New England Biolabs) pQCXIP-EGFP target vector without addition of extra bases. This procedure resulted in the generation of *FGFR4-GFP* fusion genes containing in-frame C-terminal GFP-tags.

Subsequently, Stellar competent cells were transformed with the generated *FGFR4-388Gly-GFP,* *FGFR4-KD(K504M)-GFP* or *GFP*-only vectors as recommended in the manufacturer’s protocols (In-Fusion HD Cloning Kit) and selected using ampicillin (50µg/mL). DNA from the bacterial cultures was isolated using mini- and midi-prep systems (Promega, ‎Madison, Wisconsin‎, USA) according to manufacturer’s recommendations and checked for accurate cloning of the base changes by restriction digest and sequencing using the following primers:

Forw. CMV: 5’- GCAGAGCTCGTTTAGTGAACC -3’

Rev. GFP: 5’- CTGAACTTGTGGCCGTTTAC -3’

Rev. EGFP: 5’- AAGTCGTGCTGCTTCATGTG -3’

CaCl_2_ transfection for retrovirus production

Retroviruses were generated by HEK-293 cell transformation with retroviral target vectors and helper plasmids encoding the retroviral *gag* and *pol* genes and the *VSV-G* envelope gene. In detail, 1.5x10^6^ Hek-293 cells were seeded in T25 flasks. After 24 h, the CaCl_2_ mixtures were prepared containing 5 μg DNA of the respective *FGFR4* variant plasmid, 240μM CaCl_2_ and the two helper plasmids. The DNA-CaCl_2_ mixture was added to the 2xHBS buffer and the whole mix was oxygenized. After 10min of incubation at room temperature, the mix was added dropwise to the medium of the cells. Upon 5h incubation, transfection medium was replaced by FCS-supplemented DMEM growth medium. After 72h, the supernatant containing the retroviral particles was filtrated through a cellulose acetate filter and stored in 1 ml aliquots at -80° C.

Cell transduction and genetic modification

For retroviral transduction, glioma cells were seeded to 50% confluence and incubated with the undiluted virus for 24h. Subsequently, when GFP-positivity was observed in at least 30% of the remaining cells, virus solution was replaced by fresh growth medium supplemented with puromycin (1µg/ml). Cells were continuously kept in puromycin-containing growth medium and propagated further for the generation of stable variant *FGFR4-GFP* or *GFP*-only overexpressing cell lines. Additionally, a pQCXIP vector-carrying retrovirus was used to analyze the impact of empty-vector transduction. Cell lines were checked for *Mycoplasma* contamination. Clean cells were stored in liquid nitrogen and regularly freshly thawed. GFP-positivity and –distribution was microscopically confirmed prior to every seeding. qRT-PCR, confocal microscopy and flow cytometry were performed in order to analyze for transgene positivity.

Transient FGFR4 inactivation

For transient FGFR4 inactivation, cells were incubated with adenoviruses encoding a truncated *FGFR4 (tFGFR4)*. In this *tFGFR4* molecule, the intracellular kinase domain of *FGFR4* was exchanged by a *CFP* tag ^9^. Transduction success was proven microscopically by checking for CFP or GFP expression in every experiment. Cells were seeded as described in the clonogenicity, sphere formation and proliferation assay sections below. In sphere formation experiments, cells were treated with tFGFR4 or GFP-only adenovirus immediately after seeding. One day later, cells were treated with 100moi *tFGFR4* virus or *GFP-*only adenoviruses and observed for their response towards FGFR4 inactivation over time, as described in the respective assays´ sections. Potential cytotoxic or growth limiting effects of the *GFP*-control virus was tested in virus titration assays with no effects observed up to 1,000moi (data not shown).

**Confocal microscopy**

5x10^4^ cells/ml were seeded in 300µl medium into 8-well removable Ibidi chamber slides (Ibidi, Gräfelfing, Germany). The next day, cells were washed with PBS and fixed with 4% paraformaldehyde for 10min at room temperature. Subsequently, cells were stained with wheat germ agglutinin (WGA, 5µg/ml) membrane stain and DAPI (1.4µg/ml) DNA stain for another 10min at room temperature. After another washing step, the slide was covered in vectashield (Vector laboratories). A Zeiss LSM 700 Confocal laser scanning microscope (Zeiss, Oberkochen, Germany) was used for microscopy.

**Flow cytometry**

1x10^5^ cells/ml were seeded in 2ml into 6-well plates. On the next day, cells were detached by trypsin/EDTA, washed, and subsequently diluted in 500µl FACS-PBS for measurement (BD LSR Fortessa X-20 Flow Cytometer). Respective untransduced control cell samples were included to evaluate the cells’ auto-fluorescence. Results are given as percent of GFP-positive cells from all living cells compared to auto-fluorescent controls.

qRT-PCR

Cells were seeded in 2x10^5^ cells/ml in 2ml into 6-well plates. The next day, RNA was isolated, reverse transcribed into cDNA, and SYBR qRT-PCR was performed as published previously ^7^. *RPL41* served as housekeeping gene. In case of tumor tissues from primary and patient-matched recurrent IDH wild-type GBM, RNA was extracted following the ReliaPrep RNA Tissue Miniprep System protocol (Promega). In all cases, *FGFR4* and *FGF19* mRNA expression levels were measured using Taqman probes (Thermo Fisher Scientific) with FAM/ROX qPCR Mastermix (Thermo Fisher Scientific), and *ACTB* was used as housekeeping gene. Hep3B, a hepatocellular carcinoma (HCC) model, exhibiting high FGFR4 levels and harboring an amplification of the FGFR4 ligand gene *FGF19* ^8^, was used as positive control in screening approaches. All primers and Taqman probes are given in Table S2. All RNAs were isolated three times. Results are given as indicated in the figure legends.

Annexin / PI staining

4x10^4^ cells/ml were seeded in 1ml medium into 12-well plates. The next day, cells were infected with *GFP* or *tFGFR4* adenovirus (100moi). After 24h, cells were washed with PBS and detached with trypsin/EDTA. Following another washing step, cells were stained using Annexin V-FITC and PI for 10-15min at room temperature. Annexin V- and PI-positive controls were killed by heating to 55°C for 20min prior to staining. Subsequently, cells were measured using flow cytometry (BD LSR Fortessa X-20 Flow Cytometer) and analyzed with the FlowJo Software (BD Biosciences). Experiments were performed at least twice.

Clonogenicity assay

Cells were seeded in 24-well plates and incubated for seven days. In case of transient *FGFR4* inactivation, *tFGFR4* or *GFP* adenovirus was added one day after seeding and cells incubated for another seven days. For stimulation assays, FGF19 (50ng/ml) was added the day after seeding and cells were incubated for seven days. Medium was removed and cells were fixed with methanol before they were stained with crystal violet. Plates were photographed and the pictures binarized using Fiji software. The number of black pixels was subsequently counted via *R* scripting. All experiments were performed at least three times independently.

Proliferation assay

3x10^4^ cells/ml were seeded in 500µl in 24-well plates. Cells were detached with trypsin/EDTA and the number of total, living and dead cells in every condition was quantified daily using CASY® cell counter. Experiments were performed three times in duplicates.

Filter-migration assay

1-2x10^5^ cells/ml were seeded in their serum-deprived medium in Falcon® trans-well culture inserts for 24-well plates (0.8µm pores). Serum-supplemented medium was added into the 24-well plate wells. The ability of the cells to migrate towards a nutrient gradient through a porous filter was tested after 48h of incubation. Afterwards, migrated cells were fixed and stained using crystal violet, and quantification was performed as described above (clonogenicity assay section).

Wound-healing assay

Cells were seeded in Ibidi culture inserts (Ibidi) into 24-well plates. The next day, inserts were detached from the plates, and cells wound-healing capacities were followed using live-cell microscopy. In case of growth factor stimulation, FGF19 (50ng/ml) was added shortly before starting the live-cell microscopy. Data were normalized to the scratch areas at time-point zero, and quantification was performed using T scratch software (CSE lab, Zurich, Switzerland).

Blood endothelial cell (BEC) layer invasion assay

M-cherry-expressing BEC were seeded in 300µl into 8-well-Ibidi chambers (Ibidi) in Endothelial Cell Growth Medium-2 (EBM-2, Lonza, Basel, Switzerland). Fast generation of GBM cell spheres was achieved by supplementation of 0.5% methylcellulose to the neurobasal growth medium. Accordingly, 1x10^3^ GBM cells were seeded in 150µl into 96-well ultralow attachment U-bottom plates and sphere-formation was allowed for one day. The next day, EBM-2 medium was removed, and spheres of 4 wells were collected and transferred onto BEC. Subsequently, live cell microscopy was started and disintegration of the endothelial barrier was followed every 15min. Using Fiji software, the open area in the BEC monolayer was measured over-time. Additionally, the area of the generated holes and the respective spheres were analyzed, and the ratio hole area/sphere area was calculated.

Adhesion assay

Plates were optionally coated with 10µg/ml fibronectin (FC010, Millipore) or collagen type I (C8919, Sigma-Aldrich) for 2h at 37°C. After wells were thoroughly washed with PBS, 1x10^5^ cells/ml were seeded in 100µl into 96-well plates. Cells were incubated for the indicated time periods, and subsequently non-adherent cells were washed away. Photomicrographs were taken at every indicated time point. Photomicrographs were analyzed using Fiji software by the *Particle Analyzer* plugin. Fully outspread cells were counted and quantified on the photomicrographs.

Integrin-mediated cell adhesion array

The procedure was performed as described in the manufacturer’s manual (ECM532: Merck, Darmstadt, Germany). Briefly, 1x10^6^ cells/ml were seeded in 100µl of array buffer into the respective α- and β-isoform antibody-coated plates and incubated for 30min at 37°C. Cells were fixed and stained and absorbance was measured at 560nm.

Sphere formation/re-differentiation assay

To test the sphere formation capacity of the cells, 2-4x10^3^ cells/ml, depending on the respective proliferation rates, were seeded in 24-well low-attachment plates (Corning, New York, USA) in neurobasal medium (GIBCO, life technologies, Carlsbad, CA, USA) supplemented with B27, N2, and L-Glutamine, without FCS. Cells were incubated for five days and photomicrographs were taken. In case of transient *FGFR4* inactivation, *tFGFR4* or *GFP* adenovirus was added one day after seeding and cells were incubated for four days. For stimulation assays in a 3D-culture setting, cells were seeded in neurobasal medium as described above additionally supplemented with EGF (20ng/ml). The day after seeding, FGF19 (50ng/ml) was added. After 96h, microphotographs of the growing neurospheres were taken and sphere diameter was measured using Fiji software.

To test differentiation capacity, spheres were centrifuged and resuspended in FCS-supplemented growth medium. Differentiation plasticity was tested as re-differentiation capacity of the cells after another five days. Afterwards, cells were fixed, stained, and results were gathered as described in the clonogenicity assay section.

**ATRA-induced differentiation assay**

All-trans retinoic acid (ATRA) was used to induce differentiation of GSCs as previously described ^10^. Upon ATRA supply, GSCs quickly adhere to the cell culture flask and start branching, an effect frequently observed during neural stem cell differentiation ^10^. NCH644 and NCH421K GSCs were kept in their respective medium as suggested by manufacturer’s guidelines. Spheroids were dissociated using accutase (StemPro, Thermo Fisher Scientific) and 3x10^4^ cells/ml were seeded in 24-well ultra-low attachment plates in their growth medium and inhibitors were added. After 72h, spheroids were re-plated in adhesive plates in serum-containing medium (10%) containing 10nM all-trans retinoic acid (ATRA, Sigma Aldrich) as previously described in ^10^ and drugs were added again. Differentiation capacity was observed over time and microphotographs were taken after another 72h. The maximal branching radius, indicating ATRA-induced cell differentiation of GSCs, was measured from every sphere center using Fiji software.

## Xenograft formation experiments

**Subcutaneous models:** 12-week-old female CB-17 severe combined immune-deficient (SCID) mice were purchased from Envigo Laboratories (San Pietro al Natisone, Italy). The animals were kept in pathogen-free conditions in a controlled environment including 12h-alternating light cycles, according to the FELASA guidelines. 1x10^6^ cells in 100µl serum-deprived RPMI-1640 medium were injected subcutaneously into the right flank of the animals. Animals were checked for distress development every day, and tumor size was assessed regularly by caliper measurement. Body weight was recorded every second day. Tumor volumes were calculated by (length×width^2^)/2. Animals were sacrificed upon signs of reduced wellbeing, like loss of body weight, ulceration, when the tumor exceeded a length > 20 mm in one dimension, or when no tumors were palpable six months after injection.

**Orthotopic models:** *BTL1528 GFP* or *FGFR4-KD-GFP* cells were detached using trypsin/EDTA, counted, and checked for viability before implantation into brains of NOD-scid IL2Rgnull (NSG) mice (n=5 per group). Precisely, 8 week old mice were treated with analgesia (butorphanol, 2mg/kg, subcutaneously) one hour prior to surgery. Animals were anesthetized using isoflurane gas prior to and during the intervention. The injection location was disinfected using desmoderm and 5x10^5^ cells in 10µl neurocult medium were implanted via the post-glenoid foramen. Immediately after the intervention and every following day for 72h, mice received carprofen (0.5mg/ml) subcutaneously. Animals health status and wellbeing was checked using a neurological scoring system daily and, additionally, mice were weighed twice a week. All animals were kept for 21 weeks until an endpoint was reached. Mice were sacrificed by cervical dislocation and brains were prepared as FFPE samples.

**Zebrafish xenografts:** *BTL1528 GFP* or *FGFR4-KD-GFP* cells were detached using trypsin/EDTA, counted, and checked for viability before implantation. GBM cells were transplanted into mitfa^b692/b692^; ednrba^b140/b140^ embryos at two days post fertilization^11^. Images of xenografted zebrafish were acquired on an Operetta CLS high content imager (PerkinElmer) and tumor size was quantified as described previously^11^. For quantification of migration, the areas of all tumor cells or cell clusters that had extravasated the primary tumor site per zebrafish were measured using Fiji software and summarized.

Supplementary references

1. Love MI, Huber W, Anders S. Moderated estimation of fold change and dispersion for RNA-seq data with DESeq2. *Genome Biology*. Published online 2014. doi:10.1186/s13059-014-0550-8

2. Yu G, Wang LG, Han Y, He QY. ClusterProfiler: An R package for comparing biological themes among gene clusters. *OMICS A Journal of Integrative Biology*. Published online 2012. doi:10.1089/omi.2011.0118

3. Mathieu V, Pirker C, Schmidt WM, et al. Aggressiveness of human melanoma xenograft models is promoted by aneuploidy-driven gene expression deregulation. *Oncotarget*. Published online 2012. doi:10.18632/oncotarget.473

4. Ritchie ME, Phipson B, Wu D, et al. Limma powers differential expression analyses for RNA-sequencing and microarray studies. *Nucleic Acids Research*. Published online 2015. doi:10.1093/nar/gkv007

5. Mathieu V, Pirker C, Schmidt WM, et al. Aggressiveness of human melanoma xenograft models is promoted by aneuploidy-driven gene expression deregulation. *Oncotarget*. 2012;3(4):399-413.

6. Heinzle C, Gsur A, Hunjadi M, et al. Differential effects of polymorphic alleles of FGF receptor 4 on colon cancer growth and metastasis. *Cancer Research*. Published online 2012. doi:10.1158/0008-5472.CAN-11-3654

7. Gabler L, Lötsch D, Kirchhofer D, et al. TERT expression is susceptible to BRAF and ETS-factor inhibition in BRAF V600E /TERT promoter double-mutated glioma. *Acta Neuropathologica Communications*. 2019;7(1):128. doi:10.1186/s40478-019-0775-6

8. Guagnano V, Kauffmann A, Wöhrle S, et al. FGFR genetic alterations predict for sensitivity to NVP-BGJ398, a selective Pan-FGFR inhibitor. *Cancer Discovery*. 2012;2(12):1118-1133.

9. Metzner T, Bedeir A, Held G, et al. Fibroblast growth factor receptors as therapeutic targets in human melanoma: Synergism with BRAF inhibition. *Journal of Investigative Dermatology*. 2011;131(10):2087-2095.

10. Campos B, Wan F, Farhadi M, et al. Differentiation therapy exerts antitumor effects on stem-like glioma cells. *Clinical cancer research : an official journal of the American Association for Cancer Research*. 2010;16(10):2715-2728. doi:10.1158/1078-0432.CCR-09-1800

11. Grissenberger S, Sturtzel C, Wenninger-Weinzierl A, et al. Automated compound testing in zebrafish xenografts identifies combined MCL-1 and BCL-XL inhibition to be effective against Ewing sarcoma. *bioRxiv*. Published online June 17, 2021:2021.06.17.448794. doi:10.1101/2021.06.17.448794
