# Supplementary material for: Fibroblast growth factor receptor 4 promotes glioblastoma progression: a central role of integrin-mediated cell invasiveness
Source: Acta Neuropathol Commun. 2022 Apr 28;10:65. doi: 10.1186/s40478-022-01363-2 (PMC9052585; doi:10.1186/s40478-022-01363-2)
Supplement: Supplementary file 3 — Additional file3. Supplementary Table 1 [file 40478_2022_1363_MOESM3_ESM.docx]

**Supplementary table 1**

**Antibodies used in Western blot analyses.**

| ***Antibody*** | ***Dilution*** | ***Company*** |
| --- | --- | --- |
| **Primary** |  |  |
| **FGFR4 (C-16, sc-124)** | 1:500 in 5% low-fat milk powder in TBS-T | Santa Cruz Biotechnology (Dallas, Texas, USA) |
| **P-FGFR (Thr653/654, #3471)** | 1:1,000 in 3% BSA in TBS-T | Cell Signaling Technology |
| **FAK (#3285)** | 1:1,000 in 3% BSA in TBS-T | Cell Signaling Technology |
| **P-FAK (#Tyr397)** | 1:250 in 3% BSA in TBS-T | Cell Signaling Technology |
| **Nestin (10C2, #33475)** | 1:1,000 in 3% BSA in TBS-T | Cell Signaling Technology |
| **Talin (8d4 #T3287)** | 1:1,000 in 3% BSA in TBS-T | Sigma Aldrich |
| **Integrin αV (#4711)** | 1:1,000 in 3% BSA in TBS-T | Cell Signaling Technology |
| **Integrin β1 (D6SIW)** | 1:1,000 in 3% BSA in TBS-T | Cell Signaling Technology |
| **Integrin β3 (#4702)** | 1:1,000 in 3% BSA in TBS-T | Cell Signaling Technology |
| **β-actin (A5441)** | 1:5,000 in 3% BSA in TBS-T | Sigma-Aldrich |
|  | | |
| **Secondary** |  |  |
| **mouse-anti-rabbit IgG-HRP (sc-2357)** | 1:10,000 in 1% BSA in TBS-T | Santa Cruz Biotechnology |
| **mouse IgG-HRP (GTX213111-01)** | 1:10,000 in 1% BSA in TBS-T | GeneTex (Irvine, California, USA) |
