# Supplementary material for: Fibroblast growth factor receptor 4 promotes glioblastoma progression: a central role of integrin-mediated cell invasiveness
Source: Acta Neuropathol Commun. 2022 Apr 28;10:65. doi: 10.1186/s40478-022-01363-2 (PMC9052585; doi:10.1186/s40478-022-01363-2)
Supplement: Supplementary file 4 — Additional file4. Supplementary Table 2 [file 40478_2022_1363_MOESM4_ESM.docx]

**Supplementary table 2**

**Primers for Taqman and SYBR qRT-PCR.**

| **Taqman probes** | | |
| --- | --- | --- |
| ***Probe*** | ***Cat.no.*** | ***Company*** |
| **FGFR4-FAM** | HS01106913_g1 | Applied Biosystems, Thermo Fisher Scientific |
| **FGF19-FAM** | Hs00192780_m1 | Applied Biosystems, Thermo Fisher Scientific |
| **β-actin (ACTB)-FAM** | HS99999903 | Applied Biosystems, Thermo Fisher Scientific |
| **Primer for SYBR qRT-PCR** | | |
| ***Genes*** | ***Forward primer*** | ***Reverse primer*** |
| **GFP (EGFP)** | *5‘–* ACGTAAACGGCCACAAGTTC | 5‘- AAGTCGTGCTGCTTCATGTG |
| **RPL41** | 5‘- CAAGTGGAGGAAGAAGCGA | 5‘- TTACTTGGACCTCTGCCTC |
